# A Durable Metal–Organic Framework with a Hydrophobic sp^3^‐Carbon‐Rich Nanospace Constructed from Flexible Medium‐Sized Ring Ligands

**DOI:** 10.1002/smll.202512697

**Published:** 2025-12-12

**Authors:** Junichi Usuba, Yuh Hijikata, Teppei Takahara, Shinpei Kusaka, Ryotaro Matsuda

**Affiliations:** ^1^ Department of Chemistry and Biotechnology School of Engineering and Department of Materials Chemistry Graduate School of Engineering Nagoya University Furo‐cho, Chikusa‐ku Nagoya Aichi 464‐8603 Japan; ^2^ Research Center for Net Zero Carbon Society Institutes of Innovation for Future Society Nagoya University Furo‐cho, Chikusa‐ku Nagoya Aichi 464‐8603 Japan

**Keywords:** cyclophanes, ethanol–water separation, hydrophobicity, ligand flexibility, metal–organic frameworks

## Abstract

Despite the high design freedom of metal–organic frameworks (MOFs), flexible ligands that bear sp^3^‐hybridized carbon atoms in their core are generally avoided as building blocks for MOFs because they may compromise framework stability. Herein, a 2D MOF (CBoard) is reported incorporating a dibenzocyclooctadiene with a flexible medium‐sized ring at the core as a flexible ligand. The moderate flexibility of the incorporated dibenzocyclooctadiene in CBoard reduces the strain induced by external stimuli on the coordination bonds, conferring cyclic stability upon guest adsorption–desorption. Furthermore, the sp^3^‐hybridized carbon‐rich nanospace endows CBoard with water resistance and surface hydrophobicity. This hydrophobic nanospace preferentially captures ethanol over water, highlighting its potential for applications in ethanol–water separation in dilute aqueous solutions.

## Graphical abstract

A stable 2D metal–organic framework (MOF), which incorporates dibenzocyclooctadiene with a flexible medium‐sized ring core, has been synthesized. The moderate ring flexibility enables excellent cycling stability during repeated adsorption–desorption. The sp^3^‐carbon‐rich hydrophobic nanospace provided by the medium‐sized rings shows promising performance in the separation of ethanol from aqueous solutions.

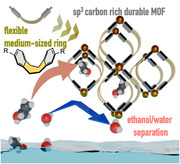

## 1 Introduction

**Figure 1:**
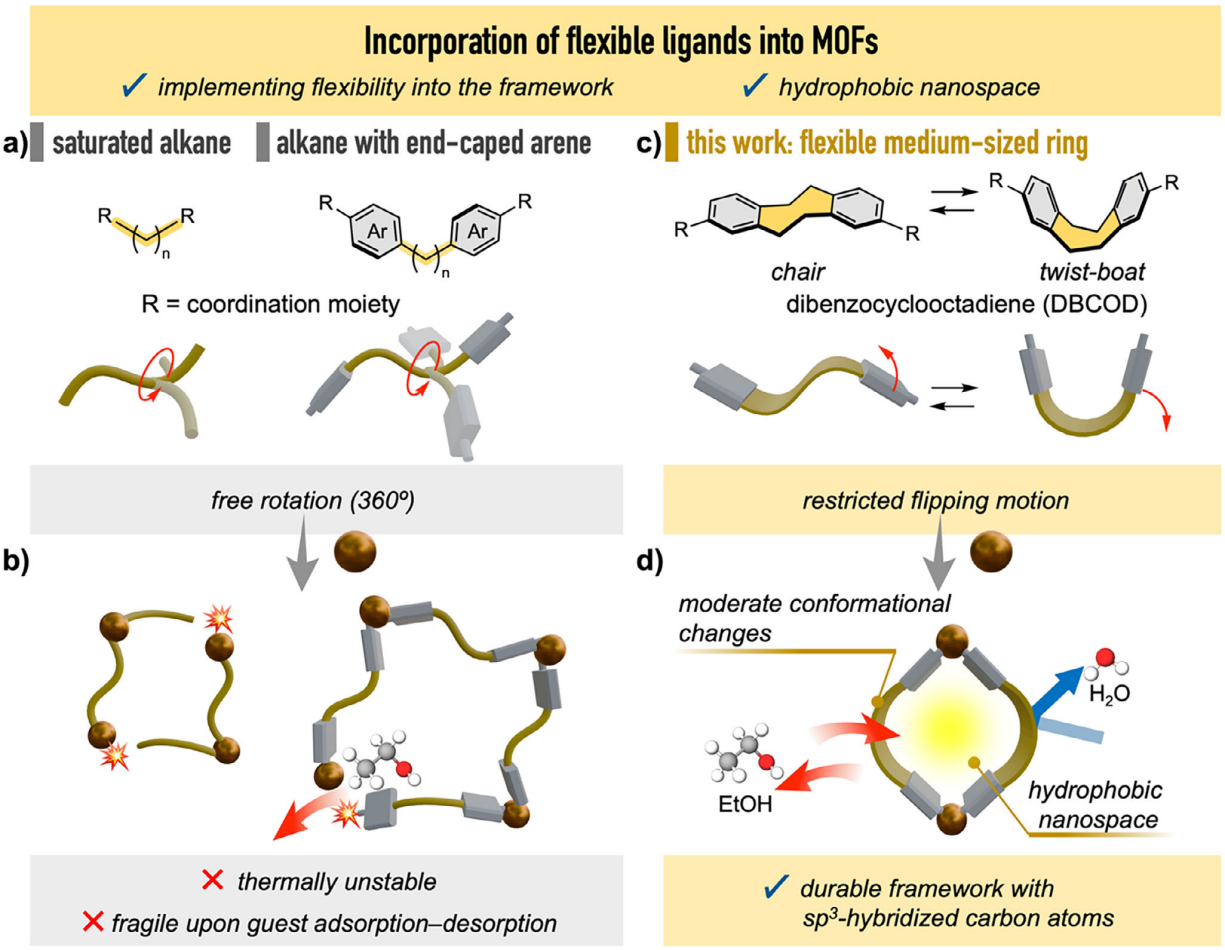
Incorporation of flexible ligands into MOFs. a) Rotational molecular motions of saturated alkane ligands and end‐capped arenes. b) MOFs that incorporate conventional flexible ligands and their limitations. c) Dibenzocyclooctadiene (DBCOD) and its directional flipping motion. d) MOFs that incorporate DBCOD.

Metal–organic frameworks (MOFs), which are formed via the self‐assembly of organic ligands and metal ions, are crystalline porous materials with a well‐defined nanospace.^[^ ^1^ ^]^ Owing to the virtually endless possible combinations of metal ions and organic ligands, novel MOFs with rich structural diversity are continuously reported. In addition, modulating the ligand length, shape, and functional groups allows controlling the pore size, guest selectivity, and responsiveness to external stimuli.^[^ ^2^, ^3^, ^4^ ^]^ Despite this high level of design freedom, most ligands incorporated into MOFs are fixed in a planar and rigid shape by sp^2^‐hybridized carbon atoms. In contrast, MOFs incorporating flexible ligands that undergo dynamic changes in molecular shape remain limited. Flexible ligands contain at least one sp^3^‐hybridized atom in the backbone.^[^ ^5^, ^6^ ^]^ For instance, saturated aliphatic ligands such as carboxylic acids^[^ ^7^, ^8^ ^]^ and adiponitrile,^[^ ^9^ ^]^ which were used in the early stages of coordination polymer research, have the potential to provide MOFs with increased flexibility^[^ ^10^, ^11^ ^]^ and hydrophobicity.^[^ ^12^ ^]^ However, the resulting MOFs are generally thermally unstable, which limits their use in applications that rely on their nanospace properties.^[^ ^13^ ^]^ In recent years, thermally stable MOFs have been prepared by incorporating ligands with flexible alkane cores end‐capped by rigid moieties such as aromatic rings (**Figure** **1**a).^[^ ^5^, ^6^, ^14^, ^15^ ^]^ However, such MOFs exhibit reduced adsorption‐surface area^[^ ^16^, ^17^ ^]^ and loss of crystallinity^[^ ^17^, ^18^, ^19^, ^20^ ^]^ upon consecutive adsorption–desorption and activation at high temperatures (Figure 1b; Table S1, Supporting Information). Furthermore, the high degree of conformational freedom of the flexible ligands leads to unexpected reaction products under the thermal conditions required for MOF synthesis.^[^ ^21^, ^22^ ^]^Consequently, the nanospace properties of MOFs surrounded by sp^3^‐hybridized carbon atoms have not been fully explored. To address this challenge, we speculated that restricting the extensive range of motion (up to 360°) around the sp^3^‐hybridized atom bonds by using dibenzocyclooctadiene (DBCOD) as a ligand motif with flexible yet directional motion could circumvent the aforementioned obstacles. The sp^3^‐hybridized carbon‐containing eight‐membered ring of DBCOD undergoes a flipping motion, switching between chair and twist‐boat forms (Figure 1c). Owing to the small energy difference (<10 kcal mol ^−1^) between these bistable and transition‐state (half‐chair form) structures, the flipping motion can occur at room temperature in solution (Figure S1 and Tables S2–S4, Supporting Information).^[^ ^23^, ^24^, ^25^, ^26^ ^]^ Thus, we hypothesized that DBCOD, with its flexible yet moderately restrained structure, could act as a robust building block to maintain framework stability (Figure 1d). Previous studies have shown that MOFs that incorporate macrocycles such as crown ethers and calixarenes exhibit framework fragility, and thus require careful activation with supercritical CO_2_.^[^ ^20^, ^26^, ^27^ ^]^ Meanwhile, medium‐sized rings at the core of ligands for MOFs have been limited to pendant substituents.^[^ ^28^, ^29^ ^]^ Herein, we report a novel two‐dimensional (2D) MOF that incorporates dibenzocyclooctadiene dicarboxylic acid (H_2_dbcoda) in its boat form. The prepared MOF (henceforth CBoard) exhibits good thermal stability comparable to that of the Cu(II) MOF family composed of rigid aromatic ligands, and retains its crystallinity upon consecutive solvent adsorption–desorption cycles. This cyclic stability is discussed in terms of the conformational change of CBoard in response to guest solvents. Furthermore, motivated by the remarkable water resistance and surface hydrophobicity of CBoard, we demonstrate its EtOH–H_2_O separation ability based on its hydrophobic nanospace.

## 2 Results and Discussion

**Figure 2:**
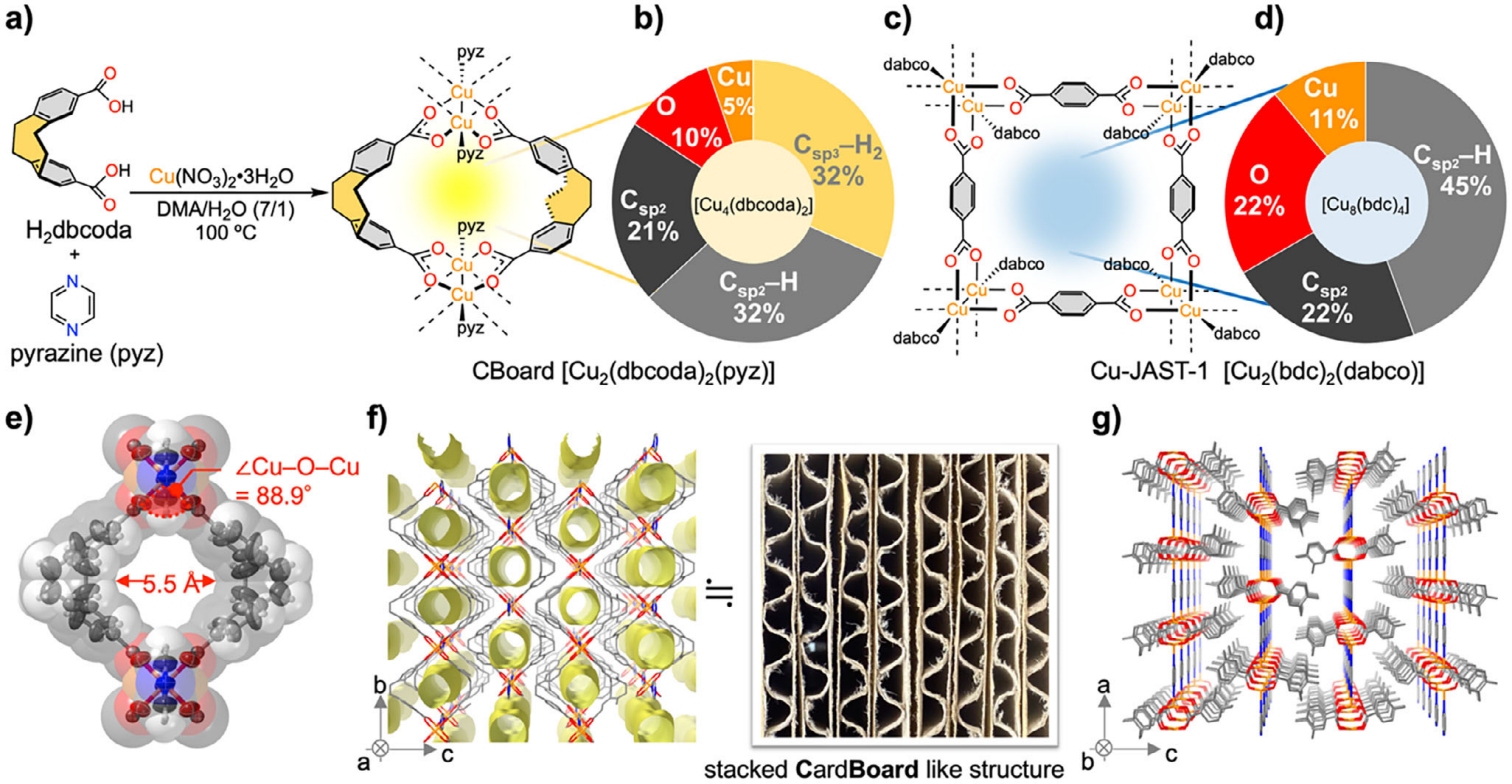
a) Synthesis of [Cu_2_(dbcoda)_2_(pyz)] (CBoard). b) Pie chart of atomic groups constituting the [Cu_4_(dbcoda)_2_] void in CBoard. c) Structure of the reference MOF Cu‐JAST‐1. d) Pie chart of atomic groups constituting the [Cu_8_(bdc)_4_] void in Cu‐JAST‐1. e) Thermal‐ellipsoid plot (50% probability) and van der Waals space‐filling model (transparent). Packing structure of CBoard viewed along the f) *a* axis and g) *b* axis.

H_2_dbcoda was synthesized from *α*,*α*'‐dibromo‐*o*‐xylene in three steps following established literature protocols.^[^ ^24^ ^]^ Using the solvothermal method, single emerald‐green crystals were obtained from a mixture of H_2_dbcoda, Cu(II) nitrate, and pyrazine (pyz) in a cosolvent of *N*,*N*‐dimethylacetamide (DMA) and H_2_O at 100 °C (**Figure** **2**a). A single‐crystal X‐ray diffraction (SXRD) analysis showed that the crystals have the composition [Cu_2_(dbcoda)_2_(pyz)], in which the paddlewheel units Cu_2_(COO)_4_ serve as the secondary building block (SBU) (Table S5, Supporting Information). One pair of dbcoda ligands is fixed as a twist‐boat and forms a small void with the partial structure [Cu_4_(dbcoda)_2_]. A comparison of the atomic groups and their ratios in the [Cu_4_(dbcoda)_2_] void with those in the [Cu_8_(dbc)_4_] void of the reference MOF Cu‐JAST‐1, which contains aromatic rigid ligands and incorporates terephthalate (dbc), 1,4‐diazabicyclo[2.2.2]octane (dabco), and Cu(II) in the [Cu_2_(dbc)_2_(dabco)] structure^[^ ^30^ ^]^ (Figure 2c), revealed that the major components of these voids are aromatic moieties (the sum of C_sp2_─H and C_sp2_), accounting for 53% in [Cu_4_(dbcoda)_2_] and 67% in [Cu_8_(dbc)_4_], respectively (Figure 2b,d). In contrast, the SBU (the sum of Cu and O) in [Cu_4_(dbcoda)_2_] is only 15%, whereas that in [Cu_8_(dbc)_4_] is 33%. In addition to the low ratio of highly polarized SBUs, [Cu_4_(dbcoda)_2_] contains a low‐polarity methylene moiety that occupies 32% of the void, which is conducive to reducing the risk of hydrolysis and creating hydrophobic nanospaces (Figure S2, Supporting Information). The size of the void in [Cu_4_(dbcoda)_2_], i.e., the distance between surfaces in the van‐der‐Waals space‐filling model, is 5.5 Å (Figure 2e). The O─Cu─O bond angle bridging the two dbcoda moieties is 88.9°, and therefore, this fragment can be regarded as an undistorted paddlewheel unit of Cu(II) containing typical carboxylate ligands.^[^ ^31^ ^]^ The void formed by two twist‐boat dbcoda ligands is bridged by pyz ligands coordinated to the axial position of the paddlewheel unit, resulting in one‐dimensional (1D) channels along the *a* axis (Figure 2f,g). The solvent‐accessible void in this 1D channel occupies 25.6% of the unit‐cell volume (567 Å ^3^; calculated using a CO_2_ probe kinetic diameter of 3.3 Å). Regarding the topology, in contrast with typical 2D MOFs, which adopt structures in which 1D channels penetrate along the normal direction of the 2D layers, the 1D channels constructed by dbcoda penetrate the layers horizontally. The topology proposed by the ToposPro program^[^ ^32^ ^]^ was classified as 4,6L44. MOFs with this topology and guest‐accessible 1D channels are rare.^[^ ^33^, ^34^, ^35^, ^36^, ^37^ ^]^ To highlight the distinctive stacked cardboard‐like structure of the [Cu_2_(dbcoda)_2_(pyz)] MOF, we named it CBoard. The guest‐accessible (100) facet has the largest area of the crystal face on the platelet crystal (Figure S3, Supporting Information).

**Figure 3:**
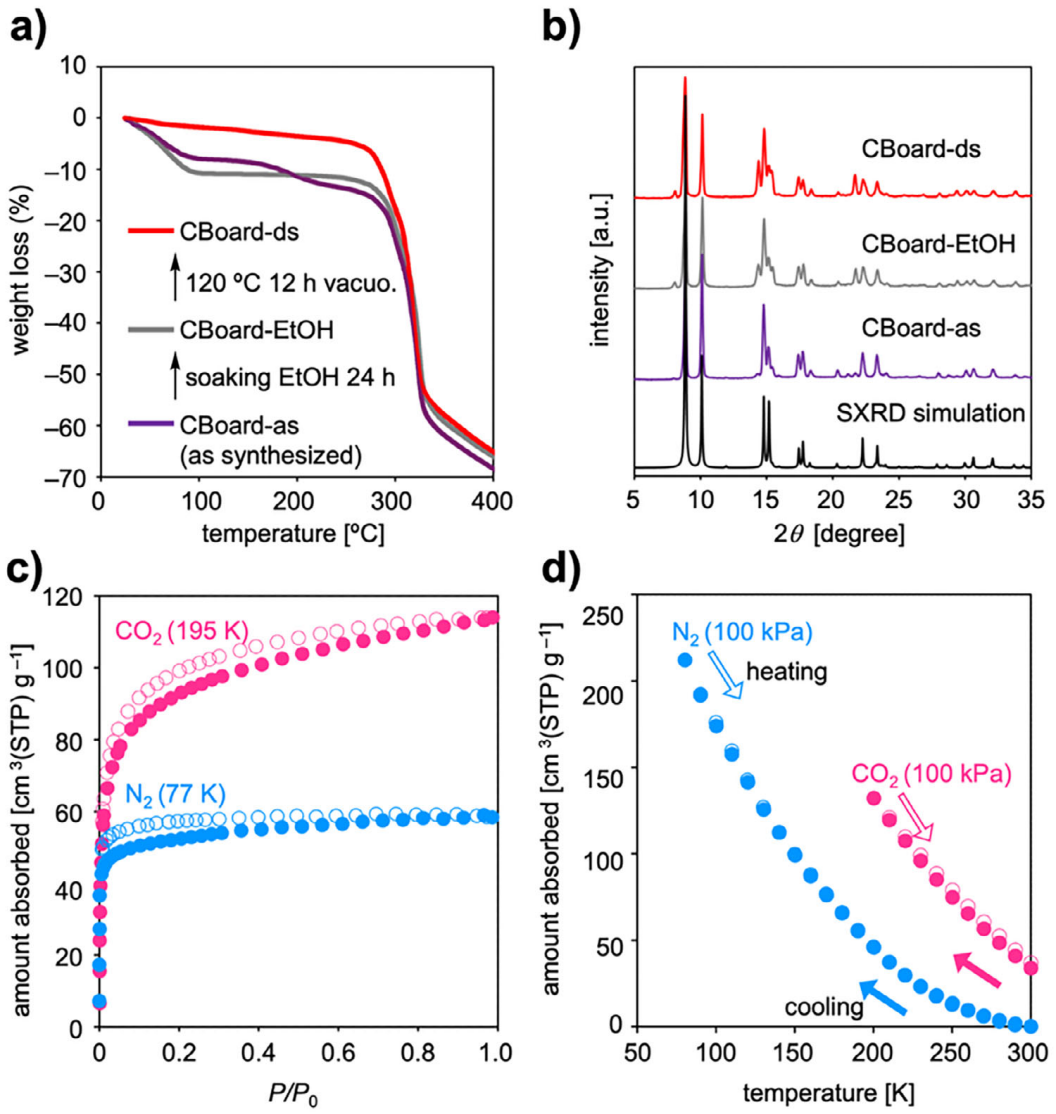
Thermal stability and gas‐adsorption properties of CBoard. a) TGA curves of CBoard‐as (purple), CBoard‐EtOH (gray), and CBoard‐ds (red). b) PXRD patterns of CBoard‐as (purple), CBoard‐EtOH (gray), CBoard‐ds (red) and simulated pattern obtained from SXRD (black). c) Adsorption isotherms of CO_2_ (195 K, pink) and N_2_ (77 K, sky blue). Closed and open circles represent the adsorption and desorption isotherms, respectively. d) Adsorption isobars of CO_2_ (pink) and N_2_ (sky blue) of CBoard‐ds at 100 kPa. Closed and open circles indicate cooling and heating processes, respectively.

The thermogravimetric analysis (TGA) of as‐synthesized CBoard (CBoard‐as) showed a two‐step weight loss in the temperature range of 30–270 °C (7% at 30–97 °C and 4% at 162–230 °C), attributable to the release of EtOH used for purification and DMA used for synthesis (**Figure** **3**a). Residual DMA trapped in CBoard was exchanged with EtOH by soaking in excess EtOH for 24 h, affording CBoard‐EtOH. To remove the guest molecules and obtain desolvated CBoard (CBoard‐ds), CBoard‐EtOH was heated at 80 °C under vacuum for 12 h. The powder X‐ray diffraction (PXRD) pattern of CBoard‐ds showed good agreement with that simulated from the SXRD data, indicating that the crystallinity was preserved during guest exchange and activation (Figure 3b). In contrast, heating to >270 °C resulted in the collapse of the framework, as evident from the broadened PXRD pattern and substantial weight loss (Figure S4, Supporting Information). Notably, despite incorporating flexible sp^3^‐hybridized carbon atoms at the backbone, the thermal stability of CBoard is comparable to that of MOFs that contain rigid aromatic ligands,^[^ ^38^ ^]^ such as Cu‐JAST‐1 (Figure S5, Supporting Information).^[^ ^30^ ^]^ The excellent stability of the CBoard framework allowed us to perform CO_2_‐and N_2_‐adsorption measurements in order to analyze the pore characteristics. CBoard‐ds showed typical type I adsorption isotherms for CO_2_ (195 K) and N_2_ (77 K). The latter was used to estimate the Brunauer–Emmett–Teller (BET) surface area (202 m^2^ g^−1^; Figure 3c). This value is considerably higher than that of previously reported 2D MOFs that bear diphenyl sulfone ligands (20 m^2^ g^−1^),^[^ ^36^ ^]^ indicating that the length of the dbcoda ligand effectively enlarges the pore size. The maximum adsorption‐capacity values for CO_2_ and N_2_ are 114 and 58 cm ^3^ g^−1^, respectively. However, the adsorption isobars at 100 kPa indicate that when the temperature gradually decreases from 300 K, the N_2_ adsorption reaches 212 cm^3^ g^−1^ at 80 K (Figure 3d). The discrepancy between the maximum N_2_ adsorption values observed in the isotherm and isobar should most likely be attributed to a pore‐blocking effect hindering guest entry at low temperature.^[^ ^39^ ^]^

**Figure 4:**
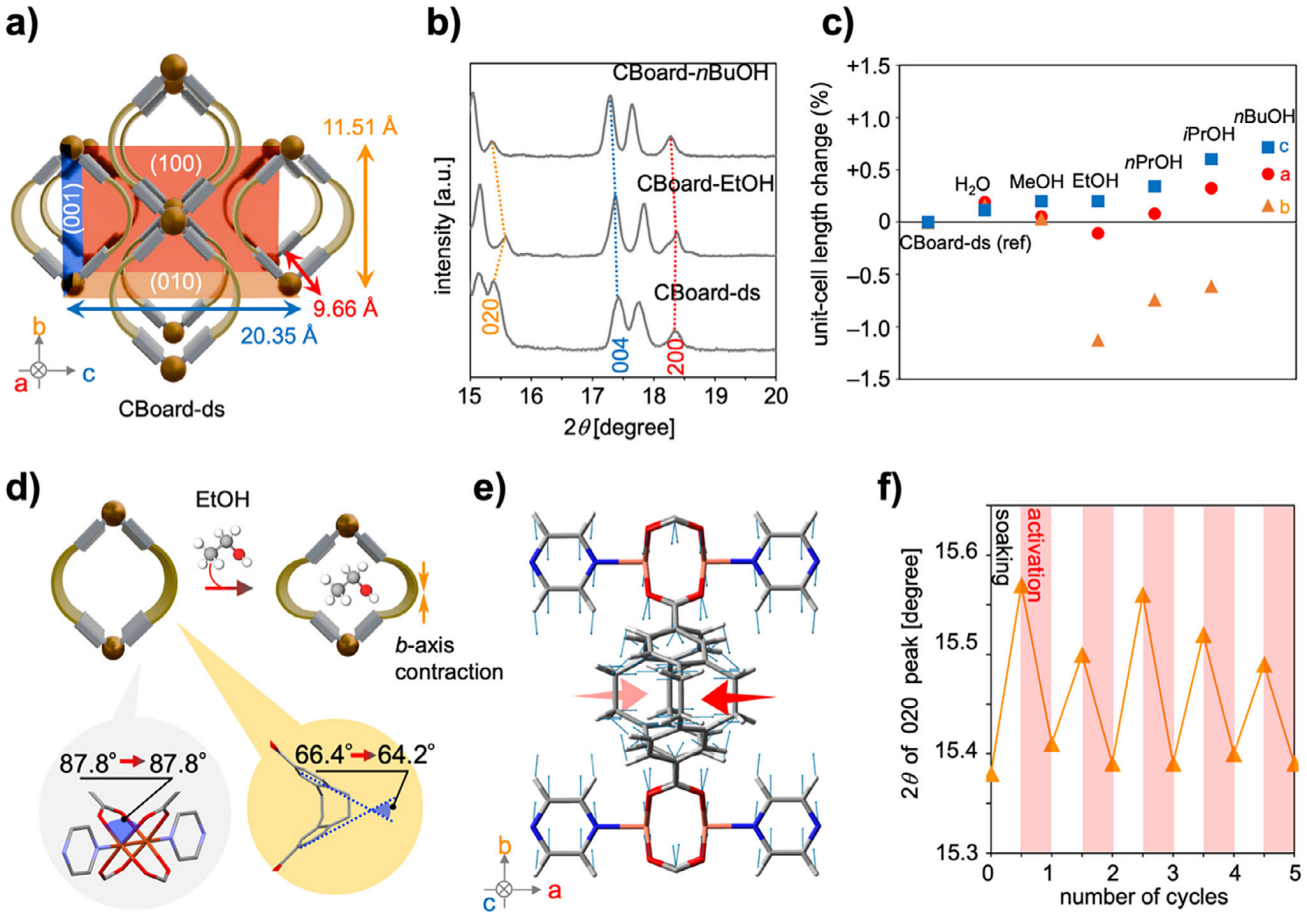
Structural changes of CBoard upon guest adsorption. a) Schematic of the simplified crystal structure of CBoard showing the (100), (010), and (001) planes. b) PXRD patterns for CBoard‐ds (bottom), CBoard‐EtOH (middle), and CBoard‐*n*BuOH (top). c) Unit‐cell length change (%) after soaking in solvents referenced to the unit‐cell length of CBoard‐ds: *a* axis (red circles), *b* axis (orange triangles), and *c* axis (cyan squares). d) Schematic and selected parameters of structural changes from CBoard‐ds to CBoard‐EtOH. e) Optimized geometry of CBoard‐ds and displacement vectors of the atom positions resulting from the conversion from CBoard‐ds to CBoard‐EtOH. Vector lengths are ten‐fold magnified for clarity. f) 2*θ *shift of the 020 peak after 30 min activation at 120 °C and 30 min soaking in EtOH.

According to previous investigations, DBCOD is flipping in solution^[^ ^24^ ^]^ but immobilized in the crystal state in a chair or twist‐boat form.^[^ ^25^ ^]^ Owing to the presence of voids in CBoard‐ds, we expected that DBCOD would be partially flexible in response to external stimuli despite being fixed in the framework. CBoard crystallizes in an orthorhombic crystal system; therefore, the cell lengths along the *a*, *b*, and *c* axes provide information about the length of the 1D channel, the stretch of the voids surrounded by dbcoda, and the interlayer distance of the 2D sheets, respectively (**Figure** **4**a). Thus, monitoring the PXRD peak positions corresponding to the (*h*00), (0*k*0), and (00*l*) planes provides insight into the structural changes of CBoard upon adsorption of guest molecules. As the obtained PXRD pattern of CBoard‐ds does not differ significantly from that simulated for CBoard‐as, the peaks at 2*θ* values of 15.38°, 17.42°, and 18.36° can be assigned to the 020, 004, and 200 reflections, respectively (Figure 4b). The changes in unit‐cell length (%) upon solvent adsorption were calculated from the 2*θ* values of these peaks (Figure 4c; Figure S6 and Table S6, Supporting Information). CBoard‐ds soaked in *n*BuOH (CBoard‐*n*BuOH) expanded along the *a*, *b*, and *c* axes compared with CBoard‐ds as the reference, and the unit‐cell volume showed a positive swelling of 1.3%. Conversely, CBoard soaked in EtOH, *n*PrOH, and *i*PrOH contracted anisotropically along the *b* axis. This tendency was most pronounced for CBoard‐EtOH, which showed a contraction of 1.1% along the *b* axis and a negative swelling of 1.0% in the unit‐cell volume. The TGA results indicated that the uptake amount of EtOH, *i*PrOH, *n*PrOH, and *n*BuOH on CBoard was 1.5–1.8 molecules per void (Figure S7 and Table S7, Supporting Information). In stark contrast, the MeOH and H_2_O uptake was small (<0.5 molecules per void) and induced minimal structural changes. Thus, CBoard undergoes structural changes within ≈1% of the unit cell depending on the guest and the adsorbed amount. To examine in detail the structural changes associated with guest adsorption–desorption, a structural optimization of CBoard‐ds and CBoard‐EtOH was conducted under periodic‐boundary conditions using the cell parameters extracted from the PXRD analysis. In the paddlewheel unit, the O─Cu─O bond angles bridging the two dbcoda ligands are 87.8° for both CBoard‐ds and CBoard‐EtOH, with a difference of <0.1° (Figure 4d). The observation that the symmetric and antisymmetric COO^–^ stretching bands (1560, 1602 cm ^−1^) in the infrared spectra of CBoard‐ds and CBoard‐EtOH are nearly identical suggests that the coordination environment surrounding the SBU is essentially preserved (Figure S8, Supporting Information).^[^ ^40^ ^]^ In contrast, the dihedral angle of the benzene rings in dbcoda is 2.2° narrower in CBoard‐EtOH (64.2°) than in CBoard‐ds (66.4°). These PXRD‐based discussions were supported by similar parameters in the single crystal structure of CBoard‐EtOH and its optimized structure (Tables S5 and S6 and Figures S9 and S10, Supporting Information). A visualization of the displacement vectors of each atom in the conformational change from CBoard‐ds to CBoard‐EtOH revealed that dbcoda undergoes a conformational relaxation that closes the boat structure while twisting along the *a* axis (Figure 4e; Movies S1 and S2, Supporting Information). Thus, conformational changes of the ligands are prioritized over distortions of the SBUs upon guest adsorption on CBoard. This strain‐relaxation mechanism can be expected to suppress the cleavage of coordination bonds during repeated adsorption–desorption, thereby enhancing framework stability. To verify this hypothesis, PXRD monitoring of CBoard was performed during repeated cycles of solvent soaking (EtOH and *n*BuOH) for 30 min and heating at 120 °C under vacuum for 30 min. For at least five cycles, the *b* and *c* axes repeatedly showed, as expected, contraction and expansion without loss of crystallinity (Figure 4f; Figures S11 and S12, Supporting Information).

**Figure 5:**
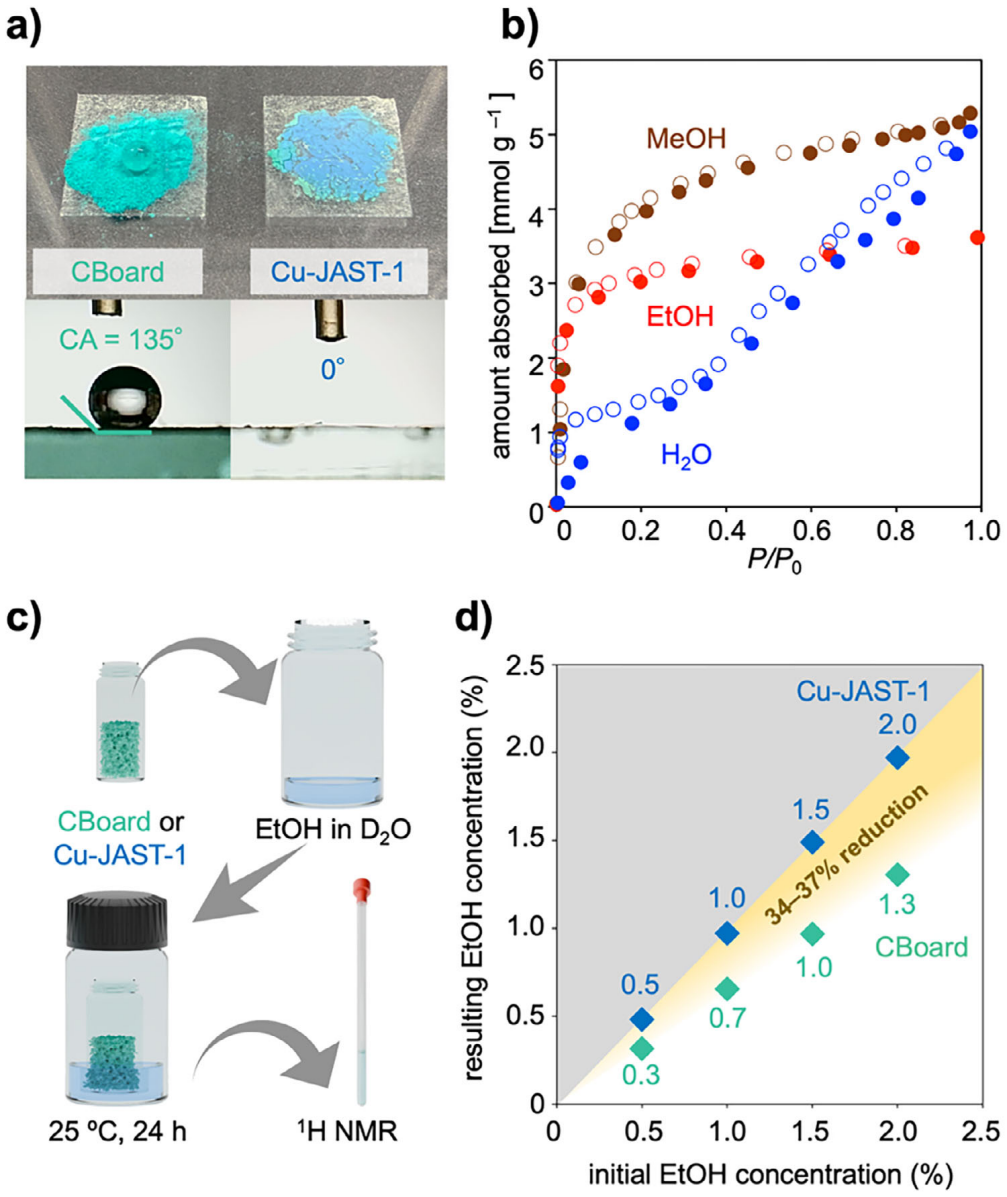
Hydrophobicity and EtOH–H_2_O separation ability of CBoard. a) Photographs of CBoard and Cu‐JAST‐1 after dropping water (top) and their corresponding contact angles (bottom). b) MeOH (brown), EtOH (red), and H_2_O (blue) vapor adsorption isotherms measured at 298 K; closed and open circles represent the adsorption and desorption isotherms, respectively. c) Schematic illustration of the experimental procedure for EtOH─D_2_O vapor separation. d) Changes in EtOH concentration during EtOH─H_2_O separation using CBoard (green) and Cu‐JAST‐1 (cyan) as adsorbents.

It should be noted here that CBoard preferentially adsorbs EtOH over H_2_O, despite EtOH having a higher kinetic diameter (4.5 vs 2.8 Å)^[^ ^41^ ^]^ and lower boiling point (78.4 vs 100 °C) than H_2_O. Except for some hydrophobic MOFs,^[^ ^42^, ^43^, ^44^ ^]^ MOFs generally adsorb H_2_O more readily than EtOH owing to their size difference. To elucidate the origin of the inverse selectivity of CBoard, the hydrophobic nanospace surrounded by sp^3^‐hybridized carbon atoms was evaluated. First, the surface hydrophobicity was assessed by measuring the contact angle (**Figure** **5**a). As shown in Figure 5a, Cu‐JAST‐1 (Figure S13, Supporting Information) absorbes water droplets into the bulk of the material, whereas droplets retained their spherical shape on the surface of CBoard. The measured contact angle of CBoard powder (135°) indicates that a hydrophobic surface of the crystal.^[^ ^42^ ^]^ In addition, even though the density of CBoard is higher than that of H_2_O (1.37 g cm ^−3^ calculated based on the SXRD data), most of the powder floated on water without loss of crystallinity for a week, further supporting its hydrophobicity (Figures S14 and S15, Supporting Information). This behavior stands in sharp contrast to that of Cu‐JAST‐1, which gradually decomposes in water.^[^ ^45^, ^46^ ^]^ To investigate the accessibility and affinity of solvent vapors to the nanospace of CBoard, H_2_O, MeOH, and EtOH vapor adsorption isotherms were measured at 298 K (Figure 5b). The CBoard shows a higher adsorption capacity for H_2_O (5.0 mmol g^−1^) than for EtOH (3.6 mmol g^−1^) at relative pressures close to 1. However, the sigmoidal curve of the H_2_O adsorption isotherm showed a type IV shape, suggesting that H_2_O─H_2_O interactions dominate over H_2_O─framework interactions.^[^ ^47^ ^]^ In stark contrast, the adsorption isotherm for EtOH exhibits a rapid uptake at even lower pressures than those for H_2_O and MeOH. These results indicate that although the nanospace of CBoard can potentially uptake both H_2_O and EtOH, it has a markedly stronger affinity for EtOH. To fully exploit the water resistance of CBoard and its ethanol affinity for separating applications, separation experiments were conducted in both vapor and liquid phases using a 15% ethanol aqueous solution, which is the typical concentration of unpurified brewed alcohol (Figure S16, Supporting Information). Simultaneous thermogravimetric‐mass spectrometry (TG‐MS) analysis revealed that ethanol uptake was greater in vapor than in the liquid phase, judging from weight loss values (at 120 °C: in vapor: −6.1%, liquid phase: −3.5%) (Figures S17 and S18, Supporting Information). Furthermore, the observation that ethanol uptake was confirmed even under a more dilute 2% ethanol aqueous solution in vapor is a noteworthy result (−3.8% weight loss). These results suggest that CBoard is particularly well suited for applications in the food industry and wastewater treatment, where ethanol must be removed from the vapor phase of dilute ethanol solutions.^[^ ^48^, ^49^ ^]^ To highlight this feature of the CBoard, its quantitative ethanol‐water separation performance with more dilute ethanol solutions was also evaluated in vapor. Specifically, a vial containing CBoard‐ds was inserted into closed vials containing a D_2_O solution of 0.5, 1.0, 1.5, and 2% EtOH, respectively, and the concentration changes were monitored using ^1^H NMR spectroscopy (Figures 5c; Figures S19–S22, Supporting Information). CBoard decreased the initial EtOH concentration by 35% (2%→1.3%), demonstrating remarkable selectivity, whereas Cu‐JAST‐1 caused almost no change in concentration (Figure 5d). This trend was observed even at an EtOH concentration as low as 0.5%, with CBoard reducing the initial EtOH concentration by 34–37%. Together with its water resistance and cycling stability against adsorption–desorption, these results showcase CBoard as a promising candidate for EtOH capture from dilute aqueous EtOH solutions.

## 3 Conclusion

We synthesized a 2D metal‐organic framework (MOF) CBoard, which incorporates a medium‐sized ring based on dibenzocyclooctadiene dicarboxylic acid (H_2_dbcoda) with sp^3^‐hybridized carbon atoms in the core as a new motif for flexible ligands. By virtue of the conformational changes of the flexible dbcoda moieties, which mitigates the risk of coordination‐bond cleavage, CBoard exhibits not only excellent thermal stability but also cyclic stability upon guest adsorption–desorption. Furthermore, CBoard demonstrates high water resistance and EtOH adsorption selectivity in H_2_O owing to its sp^3^‐hybridized carbon‐rich hydrophobic nanospace. As a result, contactless ethanol removal from low‐concentration aqueous ethanol solutions was demonstrated, which should be attractive for the food and wastewater treatment industries. These insights can be expected to open new opportunities to exploit flexible ligands containing sp^3^‐hybridized atoms in the core, which have previously been regarded as challenging building blocks, for the construction of MOFs.

## Conflict of Interest

The authors declare no conflict of interest.

## Supporting information



Supporting Information

Supplemental Movie 1

Supplemental Movie 2

## Data Availability

The data that support the findings of this study are available in the supplementary material of this article.
