# Supplementary material for: A Durable Metal–Organic Framework with a Hydrophobic sp3‐Carbon‐Rich Nanospace Constructed from Flexible Medium‐Sized Ring Ligands
Source: Small. 2025 Dec 12;22(6):e12697. doi: 10.1002/smll.202512697 (PMC12837355; doi:10.1002/smll.202512697)
Supplement: Supplementary file 1 — Supporting Information [file SMLL-22-e12697-s001.pdf]

NANO · MICRO
small

Supporting Information

for *Small*, DOI 10.1002/smll.202512697

A Durable Metal–Organic Framework with a Hydrophobic sp^3-Carbon-Rich Nanospace Constructed from Flexible Medium-Sized Ring Ligands

*Junichi Usuba, Yuh Hijikata, Teppei Takahara, Shinpei Kusaka and Ryotaro Matsuda**

Supporting Information

A Durable Metal–Organic Framework with a Hydrophobic sp^3-Carbon-Rich Nanospace Constructed from Flexible Medium-Sized Ring Ligands

*Junichi Usuba,[b] Yuh Hijikata,[b] Teppei Takahara,[a] Shinpei Kusaka,[a] and Ryotaro Matsuda[a,b]**

[a] Department of Chemistry and Biotechnology, School of Engineering, and Department of Materials Chemistry, Graduate School of Engineering, Nagoya University, Chikusa-ku, Nagoya 464-8603, Japan
[b] *Research Center for Net Zero Carbon Society, Institute of Innovation for Future Society, Nagoya university, Nagoya, 464-8301, Japan*

E-mail: ryotaro.matsuda@chembio.nagoya-u.ac.jp

Table of Contents

1. General 2
2. Computational Study 3
3. Materials and characterization methods 4–6
4. Supplementary figures and tables 7–24
5. References 25

1. General

All reagents and starting materials were purchased from commercial vendors and used as supplied unless otherwise indicated. 5,6,11,12-Tetrahydrodibenzo[*a*,*e*]cyclooctene-2,9-dicarboxylic acid (H_2dbcoda) was synthesized from α,α'-dibromo-*o*-xylene in three steps, following an established literature protocol.[1] ^{1}H NMR (300 MHz) spectra were recorded on a spectrometer (Bruker magnet system 300 MHz UltraShield), using tetramethylsilane (δ = 0.00 ppm) as an internal standard. Thermogravimetric analysis (TGA) curves were recorded on a Rigaku Thermo plus EVO2 in the temperature range of 30–500 °C under a nitrogen stream with a temperature ramp of 2 °C min^{-1}. Simultaneous thermogravimetric-mass spectrometry (TG-MS) of TGA curves were recorded on a Rigaku ThermoMass Photo in the temperature range of 30–500 °C under a helium gas flow with a temperature ramp of 20 °C min^{-1} and, mass spectrometry was measured by the electron ionization method. Microscopic images were taken using Leica M205 C. Powder X-ray diffraction (PXRD) patterns were recorded on a Rigaku model MiniFlex600 diffractometer using CuKα radiation (λ = 1.5405 Å) at room temperature with a 2θ scanning rate of 2° min^{-1}. Gas adsorption measurements were conducted on an BELSORP-MAX volumetric gas adsorption analyzer with a cryostatic temperature controller. The Brunauer–Emmett–Teller (BET) surface area was calculated using an adsorption cross section of 0.162 nm^2 (N_2). Solvent vapor sorption measurements were performed using a BELSORP-MAX X in a water bath at 298 K. Near-infrared spectra were measured using a JASCO FT/IR-4200 Spectrometer with attenuated total reflectance (ATR) method.

2. Computational Study

Density functional theory (DFT) calculations for H_2dbcoda were carried out using Becke's three-parameter hybrid exchange functional and the Lee–Yang–Parr correlation functional (B3LYP)[2] and the 6–31 + G** basis set implemented as implemented in Gaussian16 Revision B.01[3] with default thresholds and algorithms. Grimme D3-type dispersion correction with Becke-Johnson damping was included.[4] The stationary points were optimized without any symmetry assumptions and characterized by frequency analysis at the same level of theory. The transition-state optimizations were performed by single numerical differentiation (one negative frequency was identified as a convergence condition). The optimized geometries and their Cartesian coordinates are given in Supplementary Figure S1 and Tables S2–S4.

The crystal structures of CBoard-ds and CBoard-EtOH were optimized under periodic boundary conditions (PBC) using CRYSTAL17 program,[5] with reference to the experimental crystal structures of CBoard-as and CBoard-EtOH. The experimentally obtained cell parameters of CBoard-ds and CBoard-EtOH were used to optimize their crystal structures. Chemically unnecessary disordered atoms were removed from each structure prior to the calculations, while preserving the highest possible symmetry. We employed PBE functional[6] and pob-TZVP-rev2 basis set for all atoms.[7] The shrinking parameters were set to 2 and 2. The spin state of Cu paddle-wheel unit was assumed to be a triplet.

3. Materials and characterization methods

Single-crystal X-ray diffraction (SXRD)

Intensity data were collected at 100 K using synchrotron radiation (λ = 0.4130 Å) and hybrid pixel array detector (PILATUS3 X CdTe 1M) at the BL02B1 beamline of the SPring-8 with the approval of the Japan Synchrotron Radiation Research Institute (JASRI). Olex2 1.5 was used as the GUI for the analyses.[8] The structures were solved with SHELXT-2018/2[9] and refined by full-matrix least-squares methods on SHELXL-2019/3[10] and Olex2 refine 1.5. All non-hydrogen atoms were refined anisotropically, and all hydrogen atoms were placed using AFIX instructions. The modeling of the solvent molecules in CBoard-as present in the voids was analyzed using the solvent mask algorithm in olex2 due to the complex disorder and low chemical occupancy. For a void of 622 Å per unit cell, 136 residual electrons were found and assigned as 0.272 dimethylacetamide (C_4H_9NO).

SXRD was performed on CBoard-EtOH crystals, which were single crystals of CBoard-as soaked in EtOH for 24 hours, using the same measurement and analysis procedures. The modeling of some hydrogen atoms via the AFIX command did not work due to intense disorder near the symmetry center. EtOH molecules in voids were assigned using Q peaks and refined with an isotropic temperature factor because of the intense disorder.

Single-crystal facet assignment of CBoard-as was performed on a RIGAKU XtaLab P200 diffractometer with a VariMax Mo Optic with MoKα radiation (λ = 0.71073 Å), confocal monochromator and a PILATUS3 R 200K-A hybrid pixel array detector at room temperature.

Synthesis of CBoard-as

500 µL of a DMA solution (0.02 M) of H_2dbcoda and 100 µL of a DMA solution (0.1 M) of $Cu(NO_3)_2 \cdot 3H_2O$ were added to a round-bottom glass tube with an 8 mm inner diameter. After the resulting mixture was stirred on a vortex mixer, 60 µL of a DMA solution (1 M) of pyrazine and 100 µL of water were added. After stirring on a vortex mixer again, a microtube containing the resulting mixture was inserted as insert tubes into glass vials with an inner diameter of 27 mm, and the vials were sealed with a hermetic lid. After heating in an oven at 100 °C for 24 h, the vials were removed from the oven and allowed to cool to room temperature. The target products adhering to the glass walls of the microtube were peeled off using an ultrasonic cleaner. The collected crystalline powder was washed three times with EtOH using a centrifuge. Fifty of the same reaction systems of this scale were prepared, affording 159 mg of emerald-green crystalline powder as CBoard-as.

Single crystals suitable for SXRD were prepared from DMA solutions with different concentrations of ligands and metals (H_2dbcoda (0.04 M), $Cu(NO_3)_2·3H_2O$ (0.2 M) and pyrazine (2M)) in a similar manner.

Activation of CBoard

The heat-vacuum treatment of EtOH-soaked (24 h, *i.e.* CBoard-EtOH) CBoard-as was performed as the standard preparation procedure for CBoard-ds at 80 °C for 12 h.

Synthesis of Cu-JAST-1

Cu-JAST-1 was synthesized according to the following reported procedure. A MeOH (400 mL) solution of dicarboxylic acid (4.0 mmol) and formic acid (8 mL) was added to a MeOH (400 mL) solution of $Cu(CH_3COO)_2·H_2O$ (4.0 mmol), and the mixture was stirred for 2 days at room temperature. The precipitate was filtered off, and added to a MeOH/toluene (1:1, 40 mL) solution of 1,4-diazabicyclo[2.2.2]octane (dabco) (4.8 mmol), which was heated at 120 °C for 24 h in a Teflon-lined stainless-steel autoclave. The resulting powders were collected, washed with toluene, and heated at 130 °C for 12 h under dynamic vacuum for activation. The TGA curves and PXRD patterns of activated Cu-JAST-1 are shown in Figures S5 and S12.

Solvent uptake of CBoard

CBoard-solvent (solvent = H_2O, MeOH, EtOH, *n*PrOH, *i*PrOH and *n*BuOH) samples were prepared by soaking CBoard-ds in the respective solvents for 12 h, followed by gentle drying under an N_2 gas purge. The absorbed amounts were calculated from the weight loss (in the range of 30–270 °C) of CBoard-solvent heated using TGA. The cell lengths (Å) of CBoard-solvent and CBoard-ds were calculated from the 2θ values of the 020, 004, and 200 peaks in the PXRD patterns using the Bragg reflection condition.

Cycle test

For the evaluation of the CBoard cycle test, the processes of solvent soaking (either EtOH or *n*BuOH for 30 min) and heat-vacuum treatment (at 120 °C for 30 min) were conducted repeatedly, with PXRD patterns subsequently measured following each treatment.

Contact angle

The CBoard-ds and Cu-JAST-1 powders were spread onto double-sided tape attached to a glass slide, and a 4 μL water droplet was placed on the substrate to measure the static contact angle.

EtOH-H_2O separation (using TG-MS)

• vapor condition

20 mg of CBoard-ds was added to an 11 mm diameter glass vial.This glass vial was inserted into a 27 mm diameter glass vial containing 1 ml of aqueous ethanol (2, 5, 15% (v/v)). The vial with the outer cap closed was placed in an incubator at 25 °C. After 24 h, the CBoard was analyzed by TG-MS.

• liquid-phase

20 mg of CBoard-ds was added to an 11 mm diameter glass vial. To this vial was added 1 ml of aqueous ethanol (2, 5, 15% (v/v)). The vial with the outer cap closed was placed in an incubator at 25 °C. After 24 h, the aqueous ethanol was removed. The Cboard, carefully dried with a N_2 gas purge, was analyzed by TG-MS.

A schematic illustration of these procedures are shown in Figure S15.

Dilute EtOH-D_2O separation (using ^{1}H NMR)

Sodium mesitylenesulfonate (6.6 mg, 0.03 mmol) was dissolved in 3 mL of EtOH in D_2O solution (0.5, 1.0, 1.5, 2.0% (v/v)) as an internal standard reagent. 500 μL of this solution was added to a glass vial with an inner diameter of 27 mm. 200 mg of adsorbent (CBoard-ds or Cu-JAST-1), which had been heat vacuum treated (120 °C, at least 1 h), was added to another glass vial with an inner diameter of 11 mm. The vial with the outer cap closed was placed in an incubator at 25 °C. After 24 h, ^{1}H NMR of the EtOH-D_2O solution was recorded. The relative integral (I) of the methylene group (2H) of the remaining ethanol was calculated, with the methyl group (6H) of sodium mesitylenesulfonate as signal 1. From the integral I_{blank} obtained in the blank experiment without adsorbent, the resulting ethanol concentration C was calculated using equation (1) based on the initial concentration C_0.

$$C = C_0 \times I / I_{\text{blank}} \quad \text{------ (1)}$$

4. Supplementary figures and tables

Table S1. MOFs incorporating flexible arene end-capped ligands related to this work and their properties

ligand (L)	formula	BET surface area (m^2 g^{-1})	adsorbed gas	stability	reference*
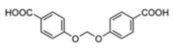	$[Cu_6(L)_3(DMF)(H_2O)]$	2101	N_2, O_2	Amorphization caused upon activation at 150 ºC.	18
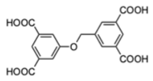	$[Cu_2(L)(H_2O)_2]$	1773 (activation at 65 ºC)	N_2	The BET SA decreased to 232 m^2 g^{-1} upon activation at 100 ºC.	16
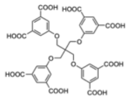	$[Cu_4(L)(H_2O)_4]$	1854 (activation at r.t.)	N_2, Ar	The BET SA decreased to 1115 m^2 g^{-1} upon activation at 120 ºC	17
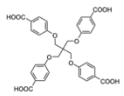	$[Cu_2(L)](H_2O)_2]$	382	N_2, H_2	Thermal decomposition at 200 ºC	19
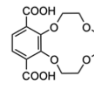	$[ZnO_4(L)_3]$	1101	N_2, CO_2, H_2		30
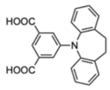	$[Cu(L)(DMA)_n(H2O)_x]$	N.A.	H_2O, D_2O		29
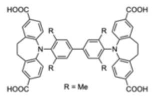	$[Zn_4O(L)_{3/2}]$	3288	N_2	Activation with supercritical carbon dioxide. R = H, $[Zn_2(L)(NMP)_2]$ was amorphized upon activation.	20
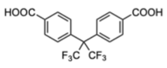	[Cu(L)(DMA)]	283	Ar,CO_2		39
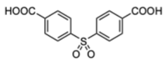	[Zn(L)DMA]	20	N_2, CO_2, H_2		28
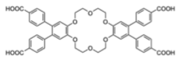	$[Zn_5(L)(OH)_2(DMF)_4]$	736	N_2, H_2	Activation with supercritical carbon dioxide. Air sensitive	27
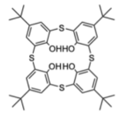	$\{[Co_4(L)_4Cl]_6(TCB)_8\}^{6-}$ TCB = 1,3,5-Tris(4-carboxyphenyl)benzene	477	N_2, H_2	Activation with supercritical carbon dioxide.	28
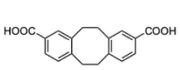	$[Cu_2(L)_2pyz]$	202	N_2, CO_2		this work

* Reference numbers correspond to the main text.

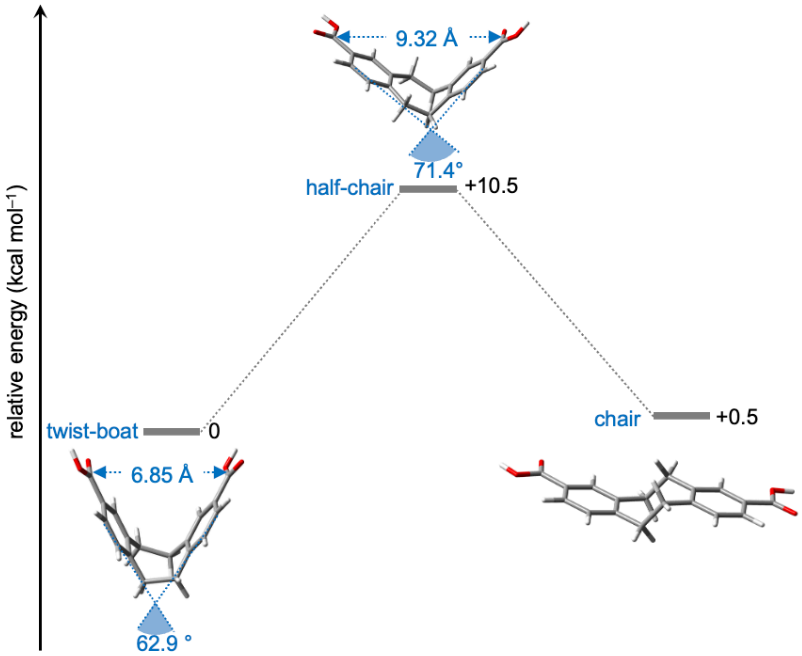

Figure S1. Optimized geometry and their relative energies (kcal mol $^{-1}$) of H_2dbcoda conformers, calculated at the B3LYP-D3/6-31+G(d,p) level of theory. Distances between the sp^2 carbons of the carboxylic groups and dihedral angles of the two benzene rings are shown in blue.

Table S2. Cartesian coordinates (Å) of the optimized geometry for H_2dbcoda (twist-boat form).

C	0.52348	-1.36966	2.17945	H	3.55404	0.32812	-1.98216
H	0.45381	-0.51802	2.86181	C	2.73388	0.79687	-0.04555
H	1.05087	-2.15864	2.73159	C	1.94439	0.33607	1.01316
C	0.88384	-3.08764	-0.25296	H	1.79595	0.99571	1.86165
H	0.82440	-3.56017	0.72993	C	-1.81534	-0.99616	-1.73419
H	1.48590	-3.75537	-0.87674	H	-1.64071	-1.38043	-2.73503
C	-0.53723	-2.98577	-0.88904	C	-2.53682	0.17938	-1.57073
H	-0.43621	-3.10079	-1.97194	H	-2.93283	0.72396	-2.42020
H	-1.13200	-3.84274	-0.54648	C	-2.75820	0.67886	-0.28431
C	-0.92200	-1.86581	1.86830	C	-2.24764	-0.01275	0.81889
H	-0.92910	-2.94998	1.73304	H	-2.41636	0.38037	1.81546
H	-1.54455	-1.66654	2.74577	C	-3.52941	1.93701	-0.14330
C	1.56469	-1.74827	-0.13767	O	-3.99201	2.57830	-1.06638
C	1.35624	-0.92751	0.99064	O	-3.68143	2.32743	1.14882
C	-1.30127	-1.70013	-0.63770	H	-4.19041	3.15364	1.11234
C	-1.52231	-1.19310	0.65941	C	3.31765	2.15519	0.06303
C	2.35741	-1.28259	-1.19207	O	3.17075	2.90527	1.00831
H	2.51154	-1.92382	-2.05573	O	4.05728	2.50443	-1.02131
C	2.94349	-0.02243	-1.15889	H	4.37977	3.40181	-0.83763

Table S3. Cartesian coordinates (Å) of the optimized geometry for H_2dbcoda (half-chair form).

H	-2.65230	-1.53600	1.14950	H	0.58814	1.23998	2.69389
C	-2.62589	-0.63124	0.55173	H	1.18002	-0.38153	2.95891
C	-2.58581	1.62450	-0.96754	C	1.77242	0.42958	1.08303
C	-1.51922	0.23434	0.70420	C	3.59048	0.66019	-1.04761
C	-3.68039	-0.39345	-0.31733	C	2.66212	-0.60408	0.79465
C	-3.65674	0.76858	-1.09791	C	1.79562	1.59349	0.29872
C	-1.49703	1.40554	-0.08443	C	2.70992	1.69567	-0.75659
H	-4.47105	0.97079	-1.78386	C	3.56776	-0.50115	-0.26573
H	-2.56555	2.52504	-1.57546	H	2.66904	-1.51091	1.39112
C	-0.55621	-0.34588	1.75132	H	2.72933	2.60274	-1.35482
H	-0.31249	-1.35541	1.39730	H	4.29559	0.74553	-1.86555
H	-1.15342	-0.49738	2.65916	C	4.48203	-1.64211	-0.51245
C	-0.49877	2.56390	-0.20829	C	-4.82846	-1.31981	-0.45403
H	-0.23571	2.60872	-1.27282	O	4.50761	-2.66893	0.13776
H	-1.07503	3.47832	-0.01872	O	-5.77085	-1.15026	-1.20284
C	0.81562	2.70192	0.56777	O	-4.73924	-2.40923	0.35234
H	1.25720	3.65226	0.24824	H	-5.53731	-2.93216	0.17158
H	0.61804	2.80569	1.63691	O	5.31711	-1.43730	-1.56348
C	0.76765	0.28526	2.19402	H	5.85782	-2.24149	-1.62689

Table S4. Cartesian coordinates (Å) of the optimized geometry for H_2dbcoda (chair form).

C	1.73551	1.19477	-0.45717	C	-4.14009	1.16315	0.07822
C	1.74673	-0.21651	-0.45456	H	-5.06950	1.70099	-0.06476
C	0.45605	1.97638	-0.63085	C	-2.94515	1.84715	0.27476
H	-0.11298	1.59396	-1.48472	H	-2.94765	2.93348	0.28608
H	0.71304	3.01117	-0.87313	C	2.95861	-0.88235	-0.26233
C	0.48217	-1.02526	-0.62850	H	2.97835	-1.96648	-0.26467
H	0.76551	-2.05562	-0.85994	C	4.15363	-0.18077	-0.06823
H	-0.08823	-0.66590	-1.49173	C	4.13981	1.21656	-0.07167
C	-0.46381	1.97033	0.63565	H	5.07107	1.75152	0.07547
H	-0.73397	3.00161	0.87857	C	2.93886	1.88668	-0.26740
H	0.11002	1.59468	1.48932	H	2.92689	2.97296	-0.27599
C	-0.44984	-1.03296	0.62712	C	5.45078	-0.86823	0.13903
H	0.11587	-0.66812	1.49117	O	6.51612	-0.30886	0.31059
H	-0.72091	-2.06715	0.85567	O	5.34598	-2.22239	0.12180
C	-1.73272	1.17191	0.46143	H	6.24714	-2.55465	0.26541
C	-1.72464	-0.24020	0.45530	C	-5.36413	-1.03598	-0.12813
C	-2.92745	-0.91832	0.26220	O	-5.41167	-2.25054	-0.14338
H	-2.95018	-2.00319	0.25868	O	-6.47385	-0.27211	-0.29938
C	-4.13289	-0.23449	0.07117	H	-7.20566	-0.89924	-0.41878

Table S5. Crystallographic datas of CBoard-as and CBoard-EtOH.

Compound	CBoard-as	CBoard-EtOH
Formula weight	182.420	154.92
Crystal system	orthorhombic	orthorhombic
Space group	*Immm*	*Immm*
Crystal color	greenish blue	greenish blue
Crystal description	block	block
Crystal size / mm^3	0.07×0.06×0.05	0.15×0.07×0.02
a / Å	9.7125(2)	9.6781(2)
b / Å	11.4739(3)	11.3742(4)
c / Å	20.3404(8)	20.3734(7)
V / $Å^3$	2266.74(12)	2242.72(12)
Z	10	10
d / g cm^{-3}	1.336	1.147
R_1 ($I > 2\sigma(I)$)	0.0890	0.1338
wR_2 (all data)	0.3460	0.4093
Goodness-of-fit	1.1183	1.842
Temperature / K	100	100
CCDC No.	2487214	2503247

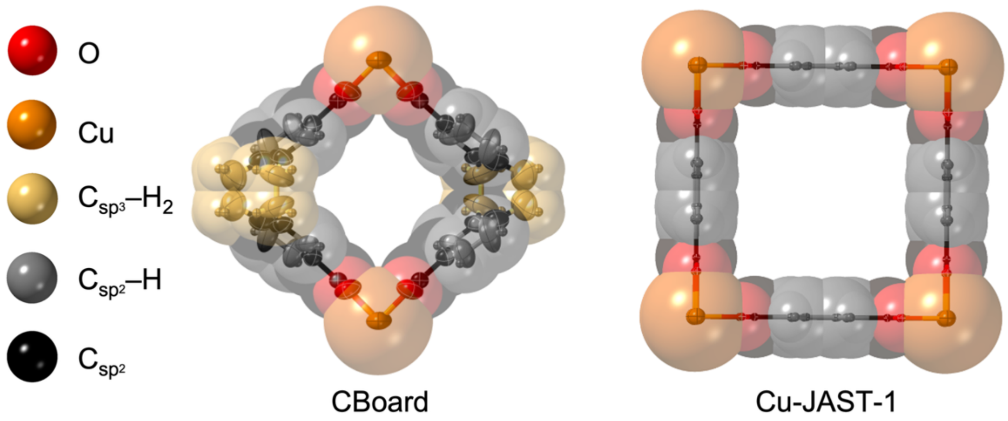

Figure S2. Thermal-ellipsoid plot (50% probability) and van-der-Waals space-filling model (transparent) for crystal structures of CBoard-as (left) and Cu-JAST-1 (right). The atomic groups were color-coded as oxygen (red), copper (orange), C_{sp^3}–H_2(yellow), C_{sp^2}–H (gray), and C_{sp^2} (black).

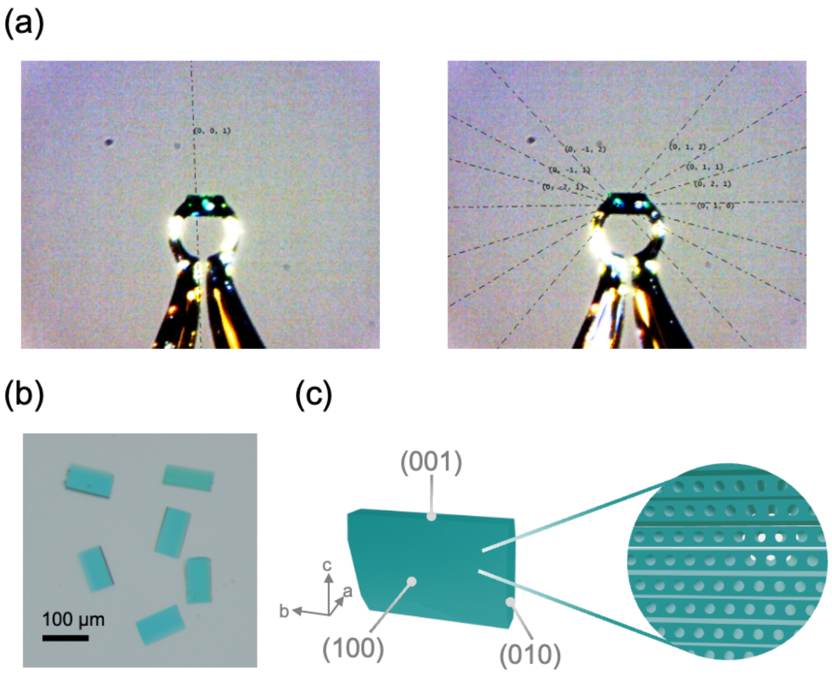

Figure S3. (a) A single crystal of CBoard-as with identified crystal facets. The dotted lines indicate the normal to the lattice planes. (b) Optical microscope image of single crystals of CBoard-as. (c) Schematic illustration of the crystal facets of CBoard-as.

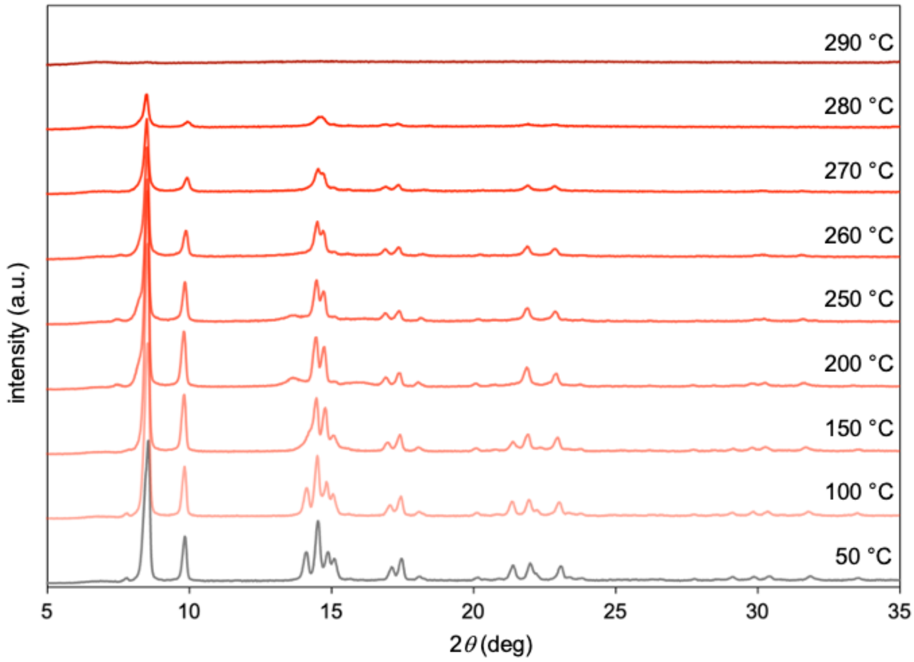

Figure S4. Variable-temperature PXRD patterns for CBoard-as

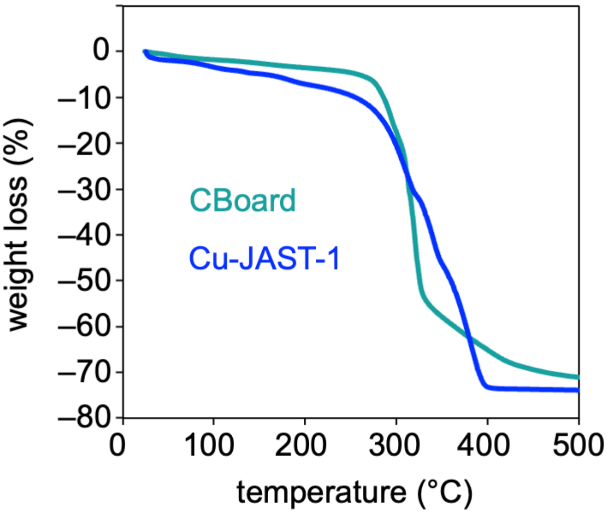

Figure S5. Thermogravimetric analysis curves for CBoard-ds (green) and Cu-JAST-1 (blue).

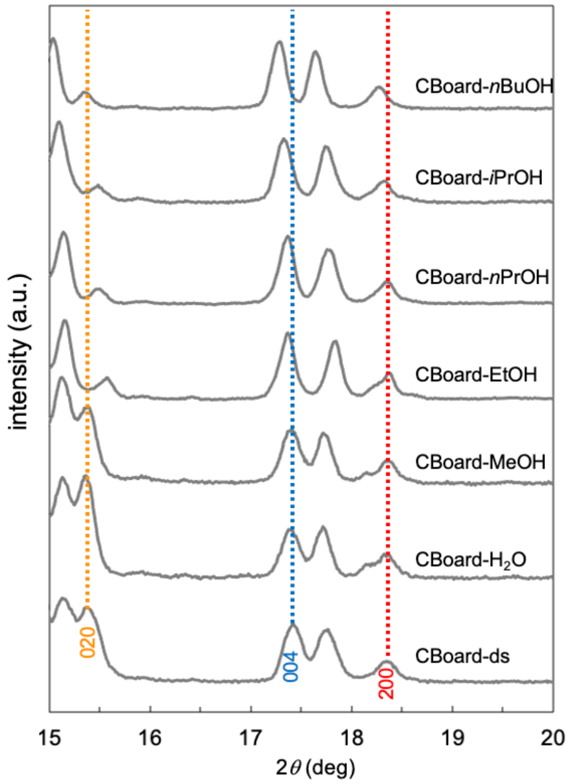

Figure S6. PXRD patterns for CBoard-ds and CBoard-solvents

Table S6. Unit cell parameters of CBoard calculated from PXRD patterns and SXRD data.

sample	*a* (Å)	*b* (Å)	*c* (Å)	*V* (Å^3)
CBoard-ds	9.659	11.513	20.353	2263.4
CBoard-H_2O	9.678	11.532	20.376	2273.9
CBoard-MeOH	9.665	11.517	20.393	2269.8
CBoard-EtOH	9.649	11.384	20.393	2240.0
CBoard-*n*PrOH	9.667	11.428	20.422	2256.2
CBoard-*i*PrOH	9.691	11.443	20.475	2270.5
CBoard-*n*BuOH	9.704	11.532	20.499	2293.8
CBoard-as (SXRD)	9.7125(2)	11.4739(3)	20.3404(8)	2266.74(12)
CBoard-EtOH (SXRD)	9.6781(2)	11.3742(4)	20.3734(7)	2242.72(12)

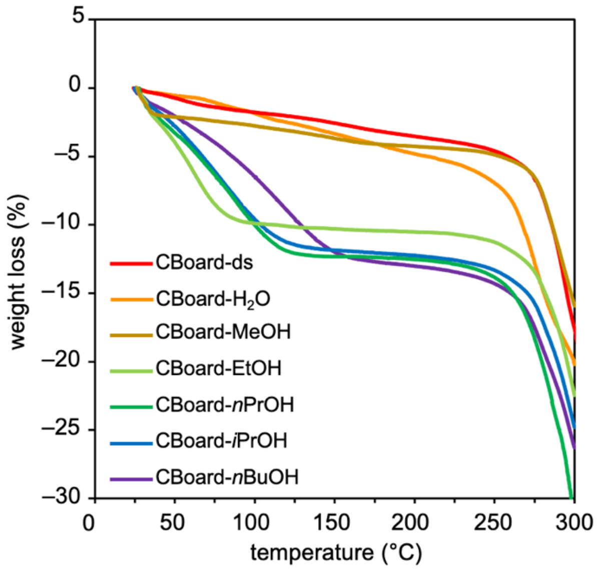

Figure S7. Thermogravimetric analysis curves for CBoard-ds and CBoard-solvents.

Table S7. Number of adsorbed solvent molecules per unit void measured from TGA.

sample	
CBoard-H_2O	0.34
CBoard-MeOH	0.52
CBoard-EtOH	1.84
CBoard-*n*PrOH	1.80
CBoard-*i*PrOH	1.74
CBoard-*n*BuOH	1.54

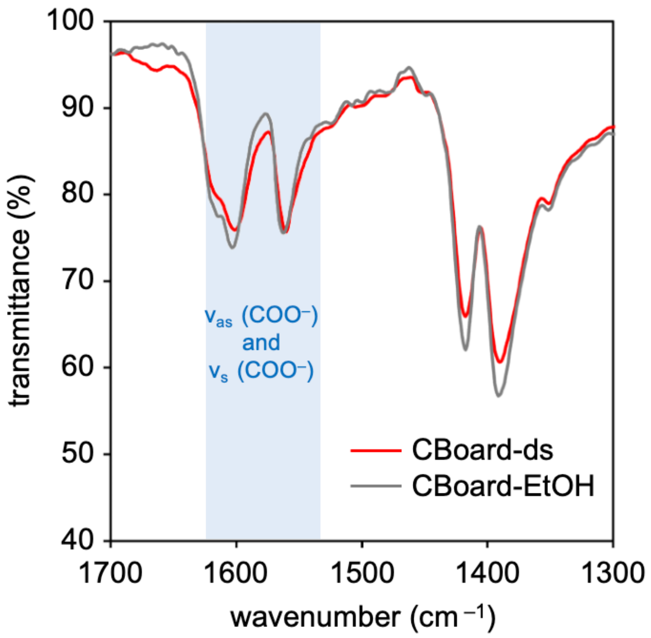

Figure S8. Infrared spectra of CBoard-ds (red) and CBoard-EtOH (gray).

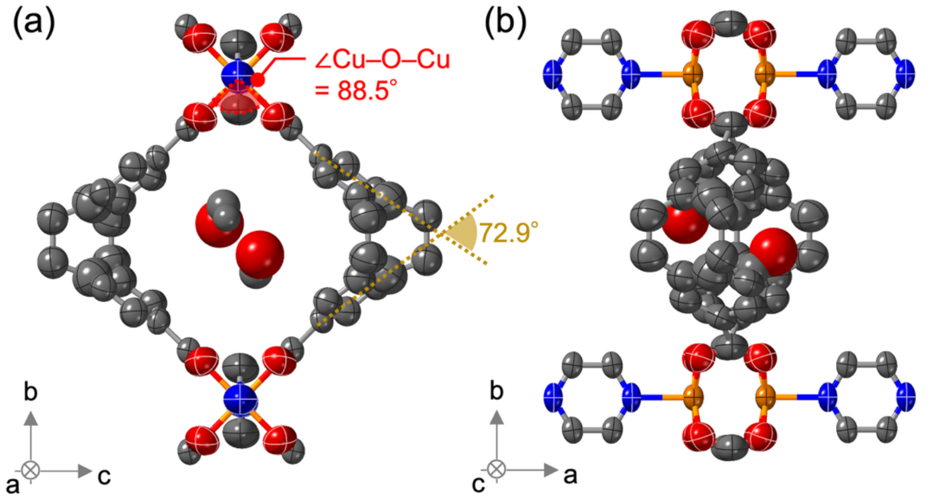

Figure S9. Crystal struture (Thermal-ellipsoid plot (50% probability)) of CBoard-EtOH viewed along the (a) *a* axis and (b) *c* axis. Hydrogen atoms are omitted for clarity.

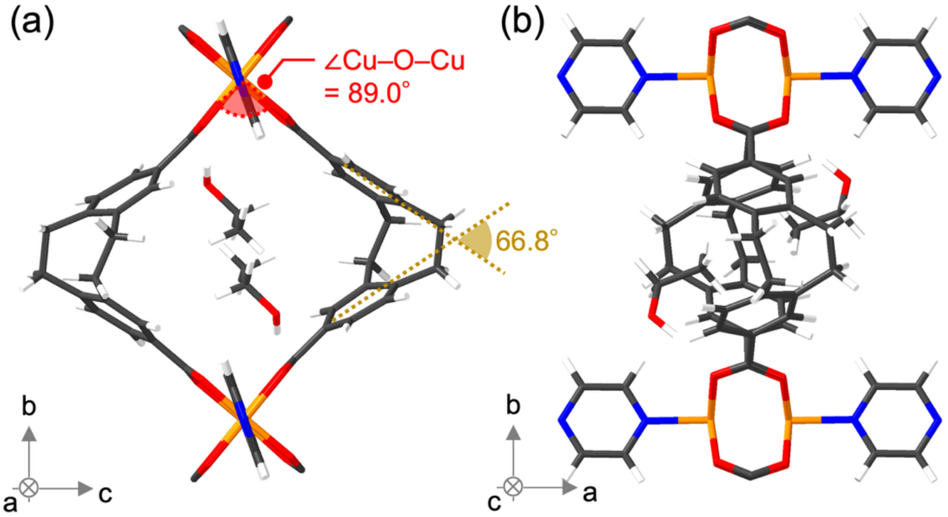

Figure S10. Theoretically optimized geometry of CBoard-EtOH corresponding to the structure determined by SXRD viewed along the (a) *a* axis and (b) *c* axis.

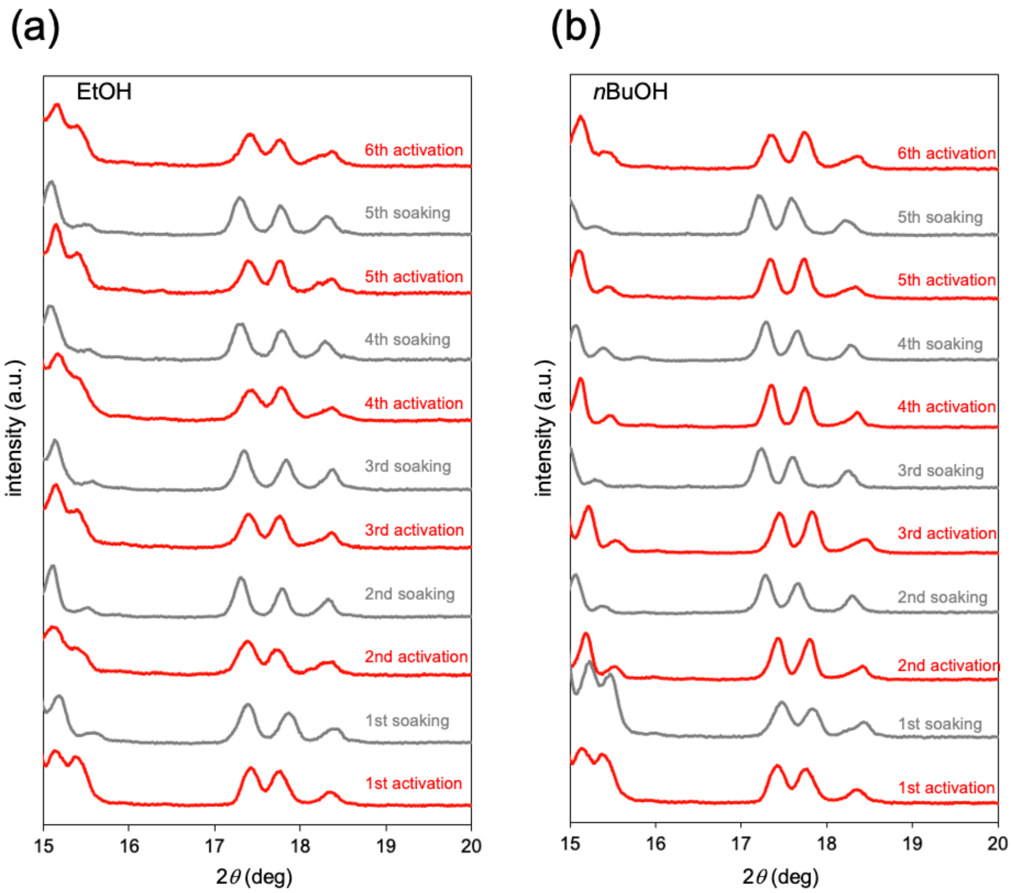

Figure S11. PXRD patterns for CBoard under the cycle test. (a) EtOH, (b) *n*BuOH.

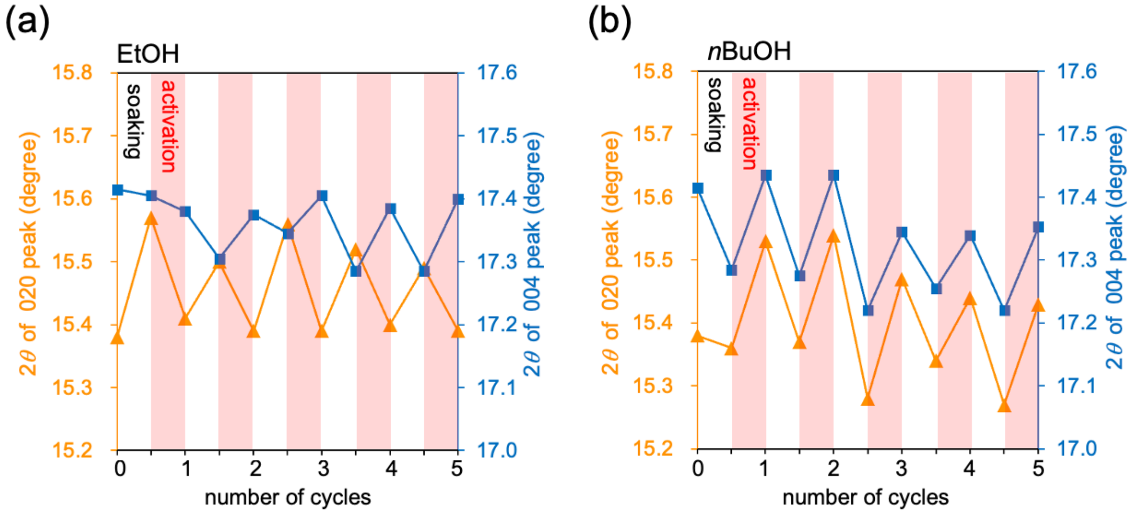

Figure S12. 2θ shifts of PXRD patterns for 020 reflection (orange triangles) and 004 reflection (blue squares) after 30 min activation at 120 °C followed by 30 min soaking in (a) EtOH and (b) *n*BuOH.

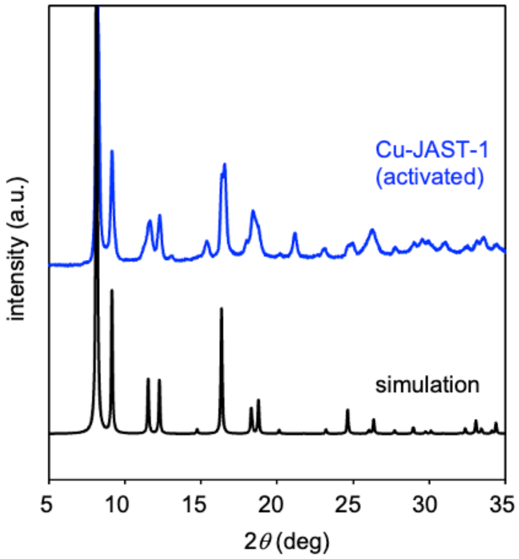

Figure S13. PXRD patterns of activated Cu-JAST-1 (top) and simulation (bottom).

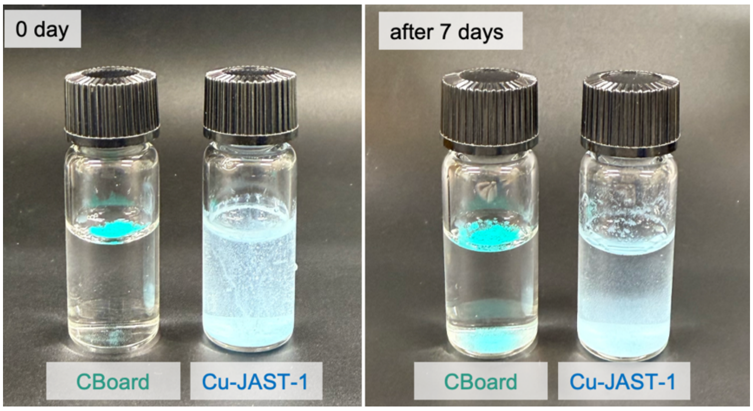

Figure S14. Photographs of CBoard soaked in EtOH and H_2O. 0 day (left) and after 7 days (right).

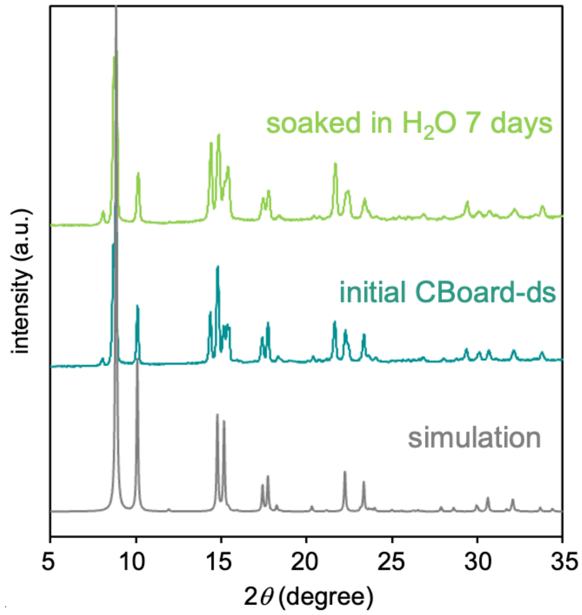

Figure S15. PXRD patterns of CBoard-ds during the water resistance test. simulation (bottom), initial CBoard-ds (bottom), and after 7 days (top).

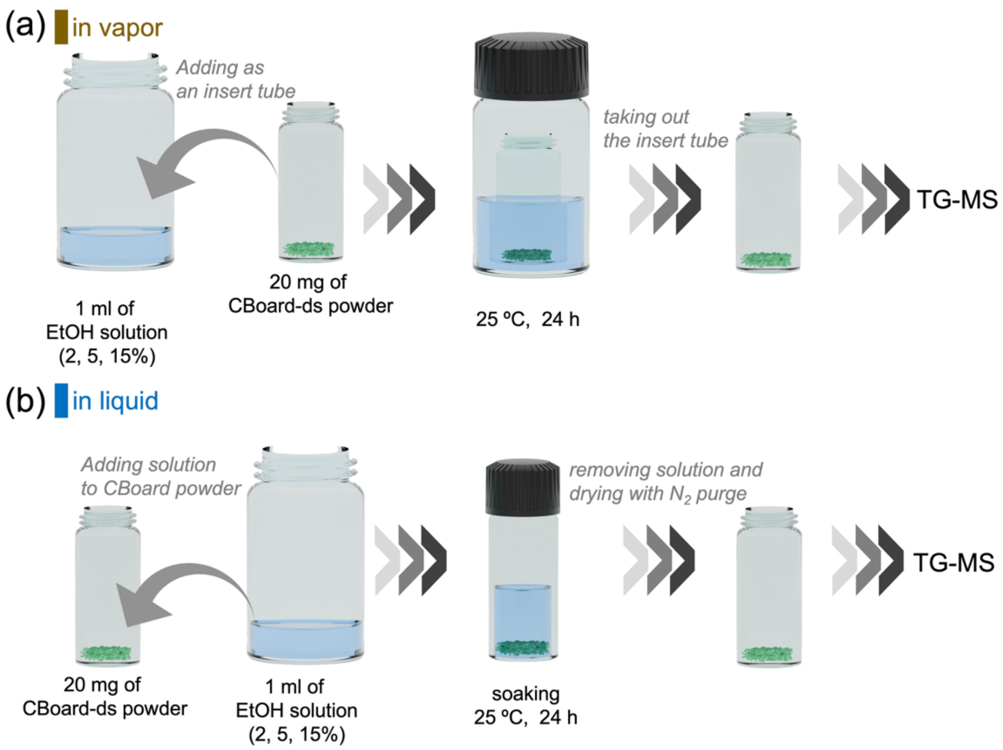

Figure S16. Schematic illustration of the experimental procedures for EtOH–H_2O separation (a) vapor and (b) liquid phase separation.

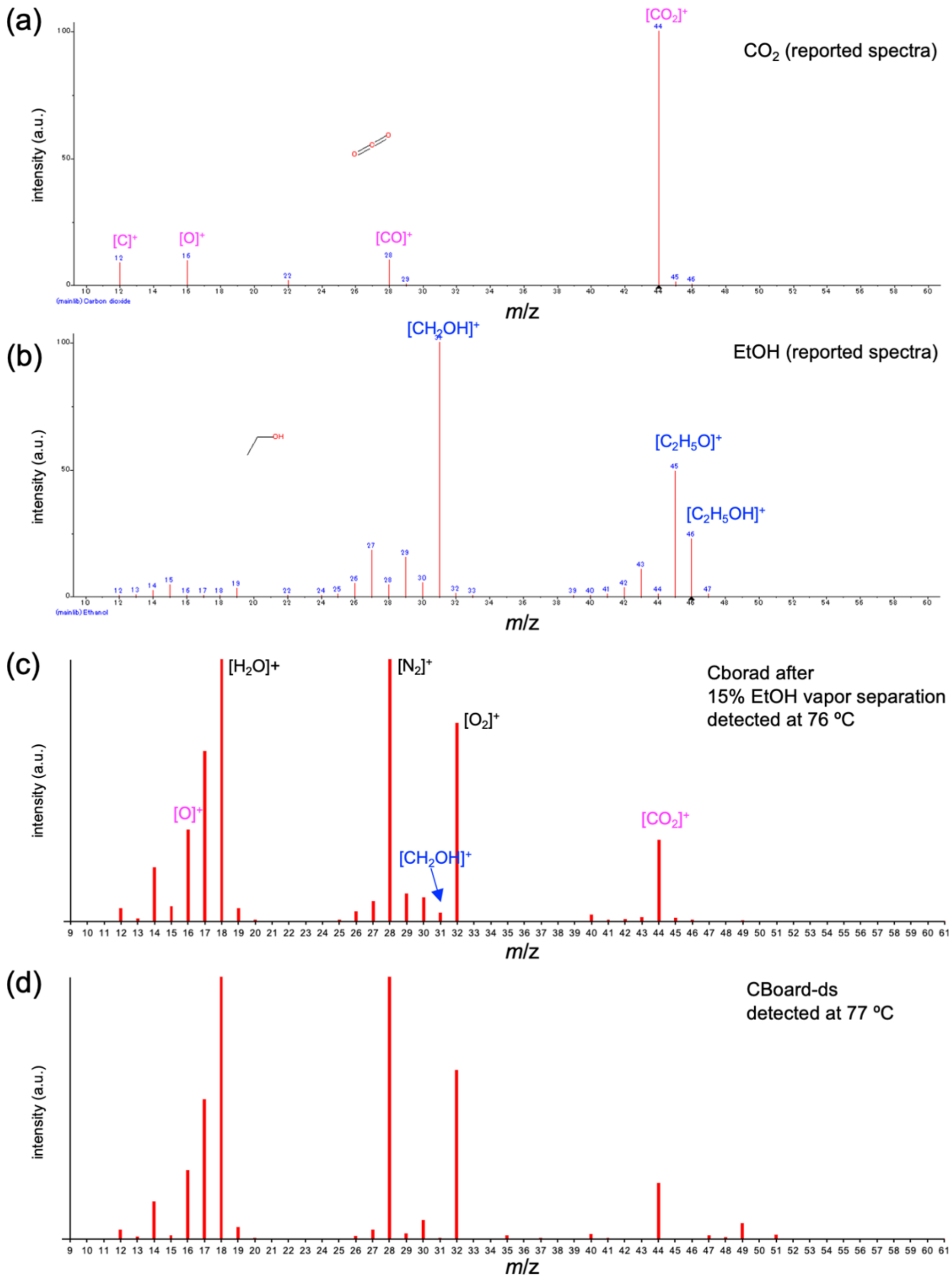

Figure S17. Reported mass spectra of (a) carbon dioxide ref[11], and (b) ethanol ref[12]. (c) mass spectra (measured by TG-MS) after 15% ethanol vapor separation experiment of CBoard, and (d) CBoard-ds.

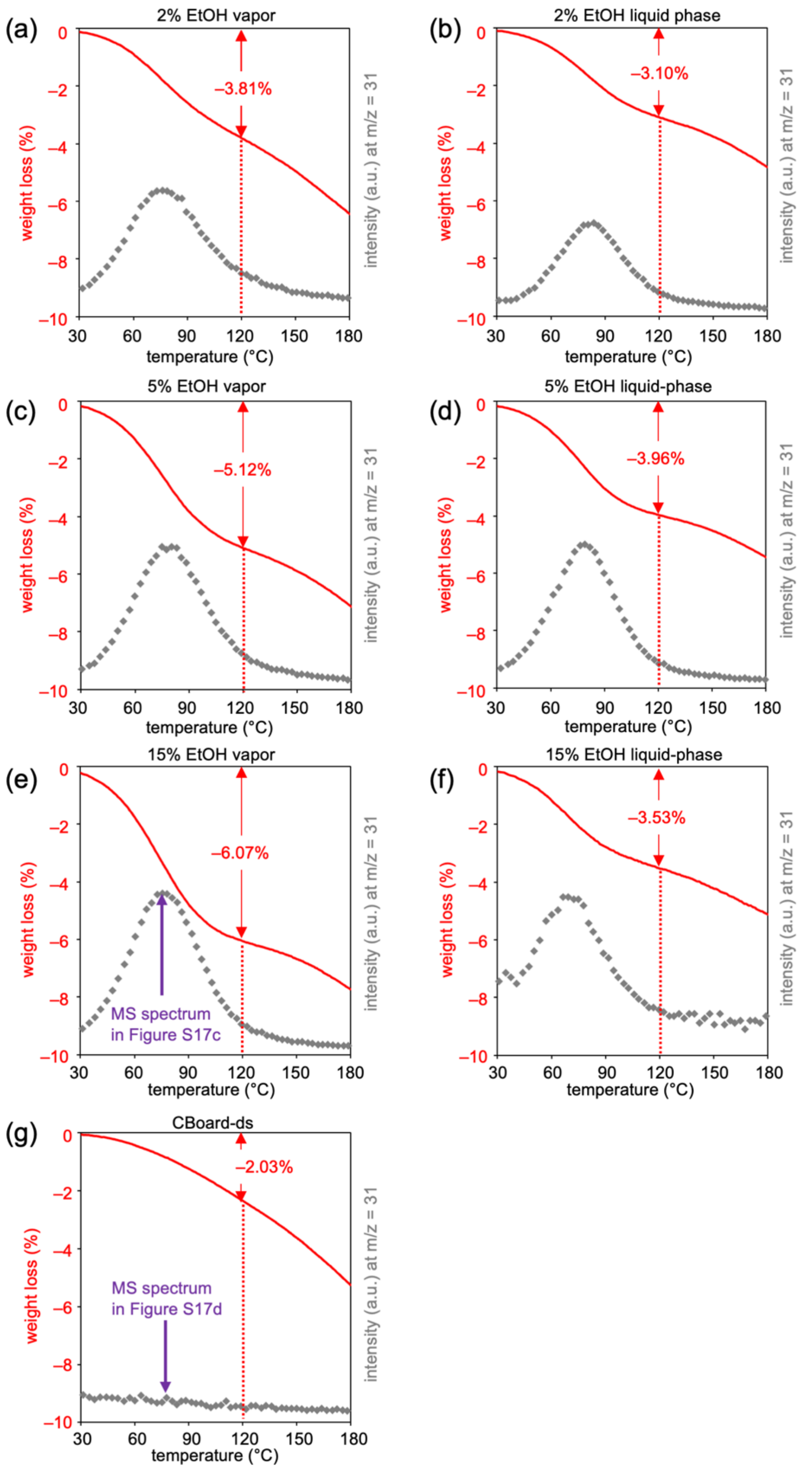

Figure S18. TG-MS of CBoard after ethanol-water separation experiment for (a) 2% EtOH vapor, (b) 2% EtOH liquid-phase, (c) 5% EtOH vapor, (d) 5% EtOH liquid-phase, (e) 15% EtOH vapor, and (f) 15% EtOH liquid-phase. (g) TG-MS of CBoard-ds. Weight loss values were shown in the range of 30–120 °C.

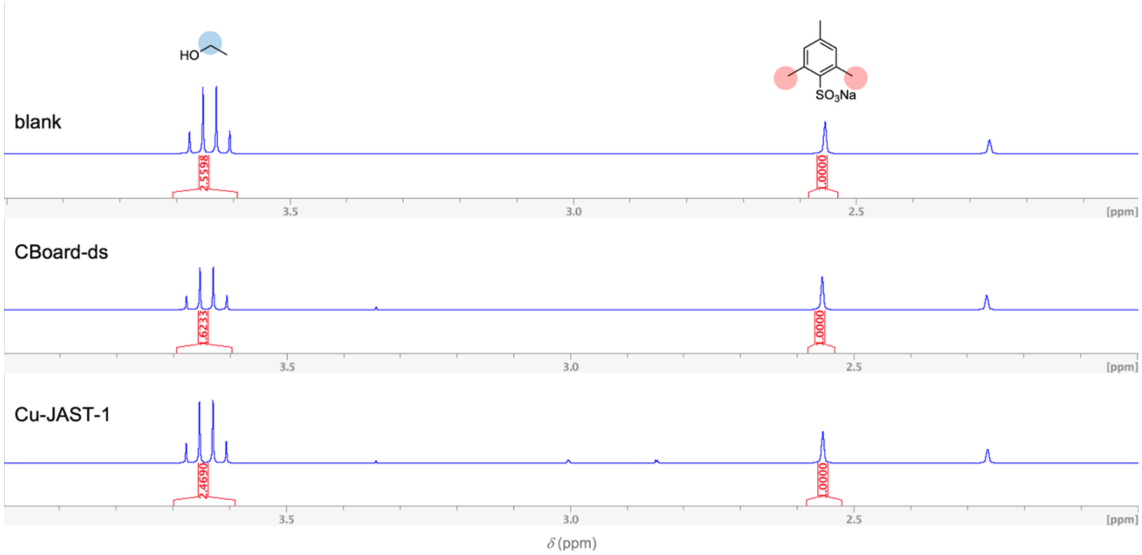

Figure S19. ^{1}H NMR spectra of the resulting solutions in an ethanol separation experiment (0.5% EtOH in D_2O). blank (top), CBoard-ds (middle), and Cu-JAST-1 (bottom).

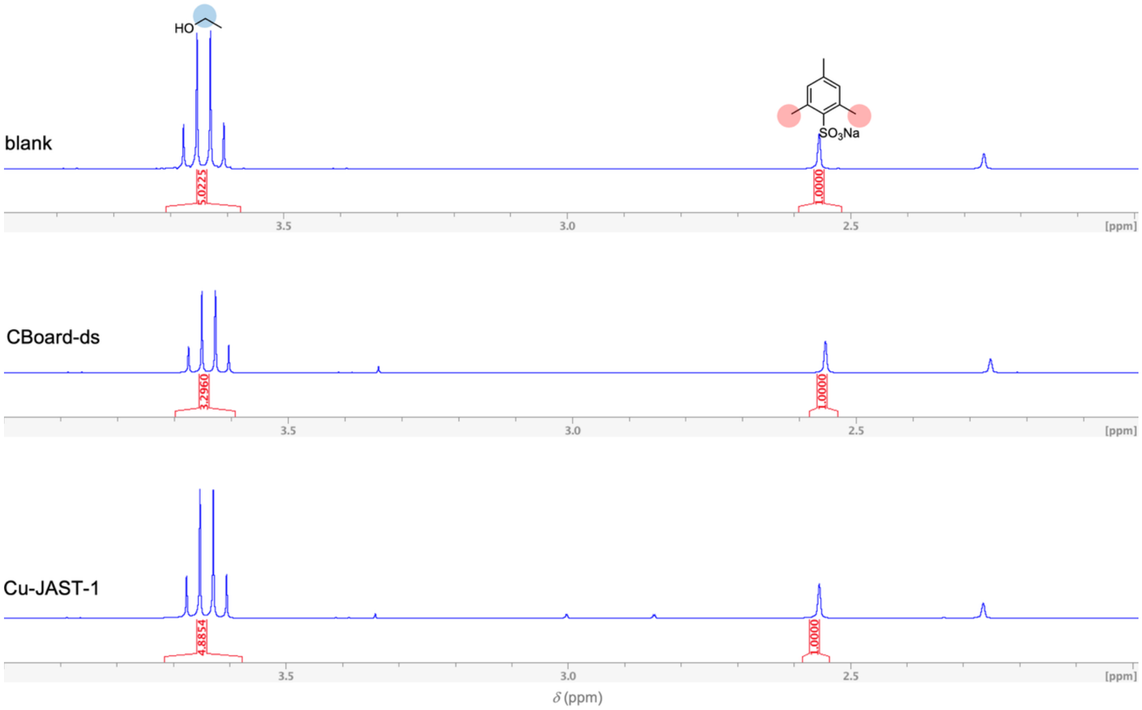

Figure S20. ^{1}H NMR spectra of the resulting solutions in an ethanol separation experiment (1.0% EtOH in D_2O). blank (top), CBoard-ds (middle), and Cu-JAST-1 (bottom).

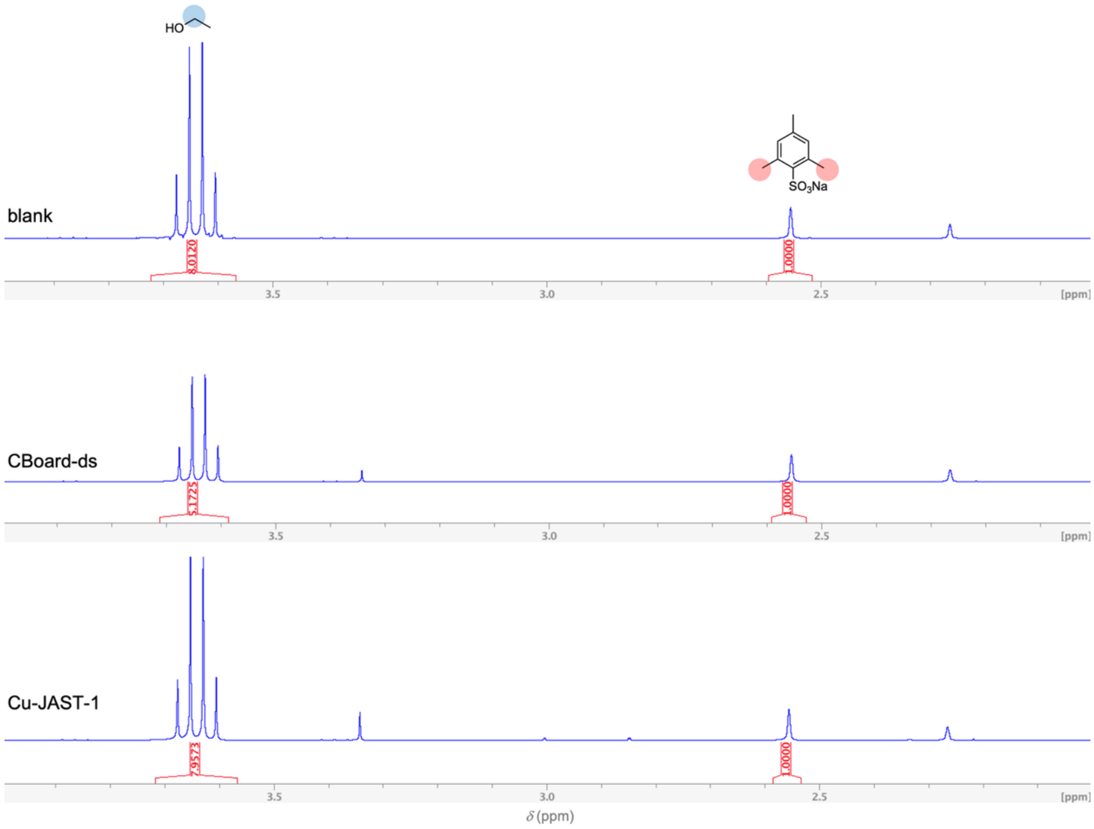

Figure S21. ^{1}H NMR spectra of the resulting solutions in an ethanol separation experiment (1.5% EtOH in D_2O). blank (top), CBoard-ds (middle), and Cu-JAST-1 (bottom).

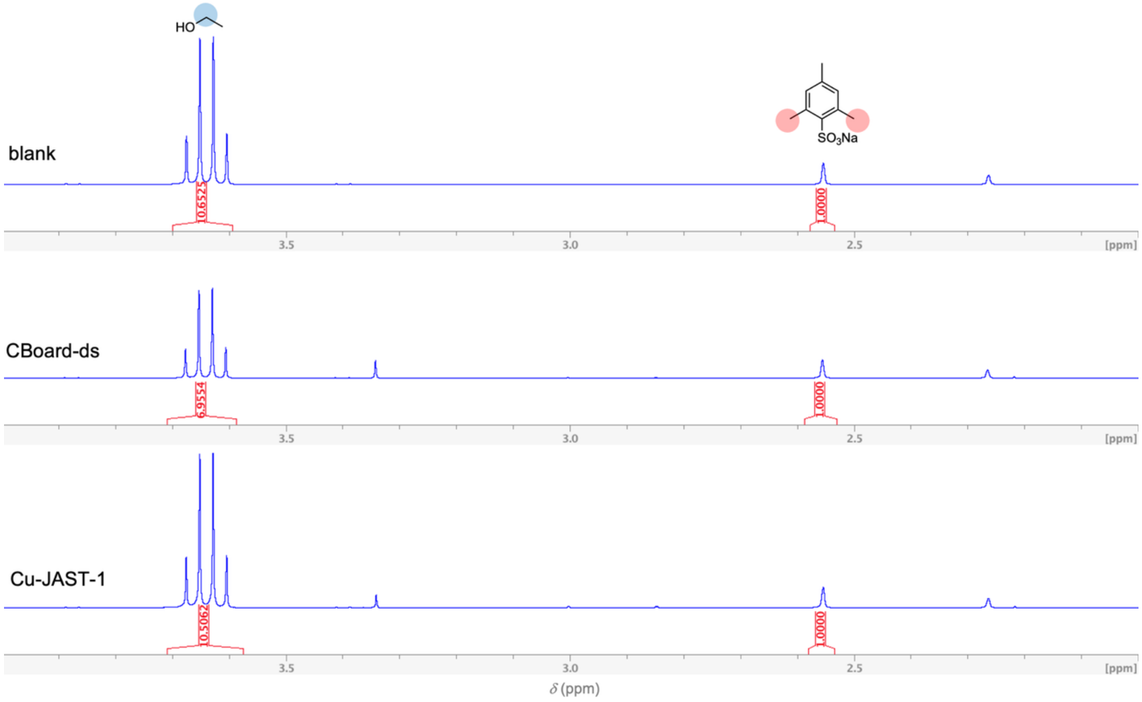

Figure S22. ^{1}H NMR spectra of the resulting solutions in an ethanol separation experiment (2.0% EtOH in D_2O). blank (top), CBoard-ds (middle), and Cu-JAST-1 (bottom).

4. References

1 Q. Sun, K. Jin, Y. Huang, J. Guo, T. Rungrotmongkol, P. Maitarad, C. Wang, *Chin. Chem. Lett.* **2021**, *32*, 1515–1518.

2 A. D. Becke, *J. Chem. Phys.* **1993**, *98*, 5648–5652.

3 Gaussian 16, Revision B.01, M. J. Frisch, G. W. Trucks, H. B. Schlegel, G. E. Scuseria, M. A. Robb, J. R. Cheeseman, G. Scalmani, V. Barone, G. A. Petersson, H. Nakatsuji, X. Li, M. Caricato, A. V. Marenich, J. Bloino, B. G. Janesko, R. Gomperts, B. Mennucci, H. P. Hratchian, J. V. Ortiz, A. F. Izmaylov, J. L. Sonnenberg, D. Williams-Young, F. Ding, F. Lipparini, F. Egidi, J. Goings, B. Peng, A. Petrone, T. Henderson, D. Ranasinghe, V. G. Zakrzewski, J. Gao, N. Rega, G. Zheng, W. Liang, M. Hada, M. Ehara, K. Toyota, R. Fukuda, J. Hasegawa, M. Ishida, T. Nakajima, Y. Honda, O. Kitao, H. Nakai, T. Vreven, K. Throssell, J. A. Montgomery, Jr., J. E. Peralta, F. Ogliaro, M. J. Bearpark, J. J. Heyd, E. N. Brothers, K. N. Kudin, V. N. Staroverov, T. A. Keith, R. Kobayashi, J. Normand, K. Raghavachari, A. P. Rendell, J. C. Burant, S. S. Iyengar, J. Tomasi, M. Cossi, J. M. Millam, M. Klene, C. Adamo, R. Cammi, J. W. Ochterski, R. L. Martin, K. Morokuma, O. Farkas, J. B. Foresman, and D. J. Fox, Gaussian, Inc., Wallingford CT, 2016.

4 S. Grimme, S. Ehrlich, L. Goerigk, *J. Comput. Chem.* **2011**, *32*, 1456–1465.

5 (a) R. Dovesi, A. Erba, R. Orlando, C. M. Zicovich-Wilson, B. Civalleri, L. Maschio, M. Rérat, S. Casassa, J. Baima, S. Salustro and B. Kirtman, *Wiley Interdiscip. Rev. Comput. Mol. Sci.*, **2018**, *8*, e1360. (b) R. Dovesi, V. R. Saunders, C. Roetti, R. Orlando, C. M. Zicovich-Wilson, F. Pascale, B. Civalleri, K. Doll, N. M. Harrison, I. J. Bush, P. D'Arco, M. Llunell, M. Causà, Y. Noël, L. Maschio, A. Erba, M. Rerat and S. Casassa, CRYSTAL17 User's Manual (University of Torino, Torino, 2017).

6 P. Perdew, K. Burke, M. Ernzerhof, *Phys. Rev. Lett.* **1996**, *77*, 3865–3868.

7 D. Vilela Oliveira, J. Laun, M. F. Peintinger, T. Bredow, *J. Comput. Chem.* **2019**, *40*, 2364–2376.

8 O. V. Dolomanov, L. J. Bourhis, R. J. Gildea, J. A. K. Howard, H. Puschmann, *J. Appl. Crystallogr.* **2009**, *42*, 339–341.

9 L. Krause, R. Herbst-Irmer, G. M. Sheldrick, D. Stalke, *J. Appl. Crystallogr.* **2015**, *48*, 3–10.

10 G. M. Sheldrick, *Acta Crystallogr. B* **2015**, *71*, 3–8.

11 R. A. Hively, R. E. Hinton, *J. Chromatogr. Sci.* **1968**, *6*, 203–217.

12 Wanakhachornkrai, *Food Chem.* **2003**, *83*, 619–629.
