# Supplementary figures and images for: A Durable Metal–Organic Framework with a Hydrophobic sp3‐Carbon‐Rich Nanospace Constructed from Flexible Medium‐Sized Ring Ligands
Source: Small. 2025 Dec 12;22(6):e12697. doi: 10.1002/smll.202512697 (PMC12837355; doi:10.1002/smll.202512697)

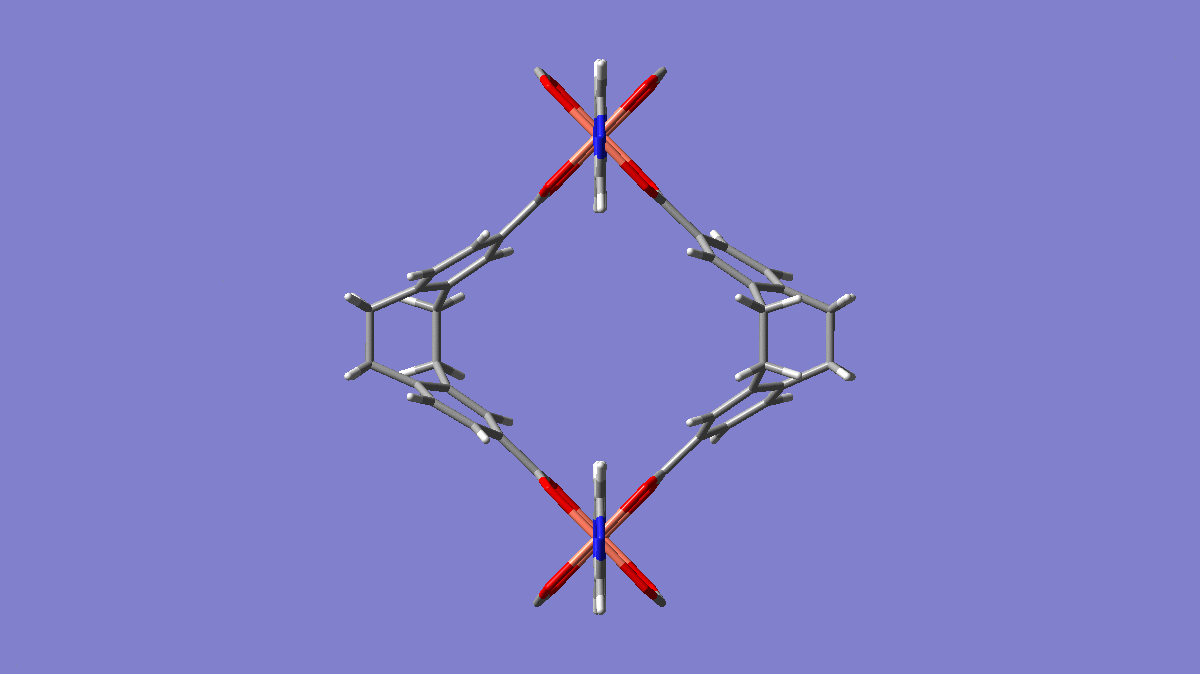

Supplement: Supplementary file 2 — Supplemental Movie 1 [file SMLL-22-e12697-s003.gif]

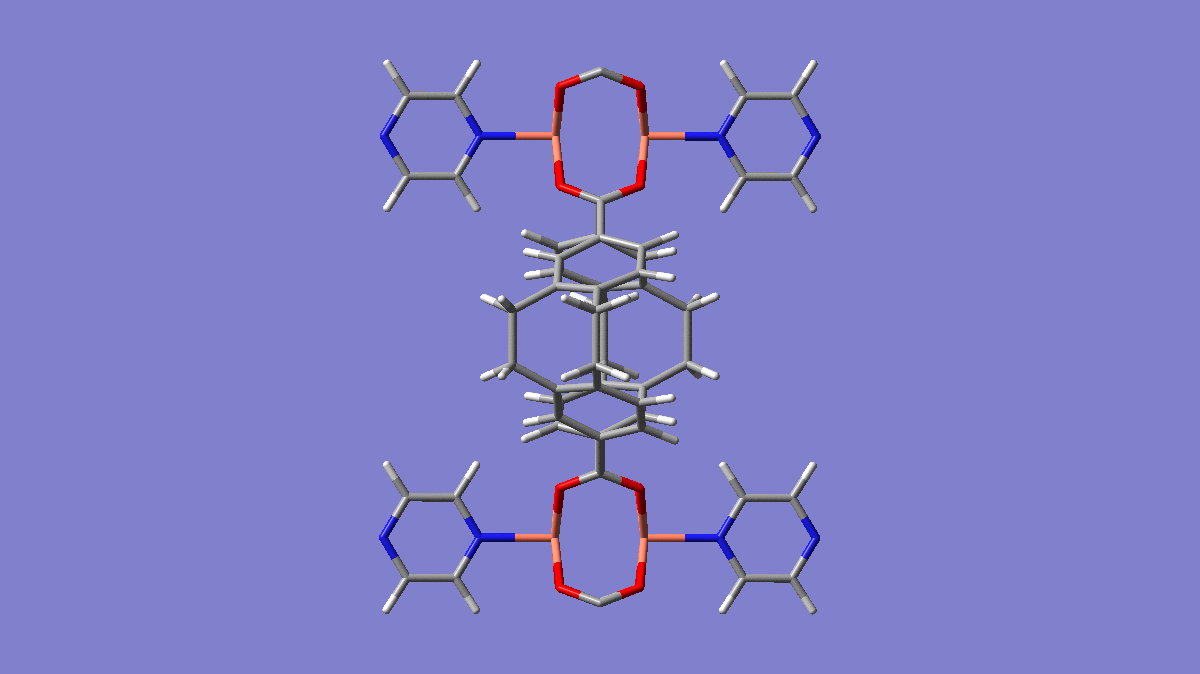

Supplement: Supplementary file 3 — Supplemental Movie 2 [file SMLL-22-e12697-s002.gif]
